# Allograft inflammatory factor-1-like is not essential for age dependent weight gain or HFD-induced obesity and glucose insensitivity

**DOI:** 10.1038/s41598-020-60433-4

**Published:** 2020-02-27

**Authors:** Dippal Parikh, Dario F. Riascos-Bernal, Lander Egaña-Gorroño, Smitha Jayakumar, Vanessa Almonte, Prameladevi Chinnasamy, Nicholas E. S. Sibinga

**Affiliations:** 1 Albert Einstein College of Medicine, Wilf Family Cardiovascular Research Institute, Department of Medicine (Cardiology), and Department of Developmental and Molecular Biology. 1300 Morris Park Avenue, Bronx, New York, 10461 USA; 2 Present Address: Diabetes Research Program, Division of Endocrinology, Diabetes and Metabolism, Department of Medicine, NYU Langone Medical Center, New York, NY 10016 USA

**Keywords:** Metabolism, Metabolic disorders, Obesity, Preclinical research

## Abstract

The allograft inflammatory factor (AIF) gene family consists of two identified paralogs – *AIF1* and *AIF1-like* (*AIF1L*). The encoded proteins, AIF1 and AIF1L, are 80% similar in sequence and show conserved tertiary structure. While studies in human populations suggest links between AIF1 and metabolic diseases such as obesity and diabetes, such associations with AIF1L have not been reported. Drawing parallels based on structural similarity, we postulated that AIF1L might contribute to metabolic disorders, and studied it using mouse models. Here we report that AIF1L is expressed in major adipose depots and kidney but was not detectable in liver or skeletal muscle; in notable contrast to AIF1, AIF1L was also not found in spleen. Studies of AIF1L deficient mice showed no obvious postnatal developmental phenotype. In response to high fat diet (HFD) feeding for 6 or 18 weeks, WT and AIF1L deficient mice gained weight similarly, showed no differences in fat or lean mass accumulation, and displayed no changes in energy expenditure or systemic glucose handling. These findings indicate that AIF1L is not essential for the development of obesity or impaired glucose handling due to HFD, and advance understanding of this little-studied gene and its place in the AIF gene family.

## Introduction

The founding member of the AIF gene family, AIF1 (also called ionized binding adapter, or IBA1), has been described as an interferon (IFN)-γ responsive pro-inflammatory molecule^1^. Interestingly, AIF1 has also been linked to metabolic conditions in several human populations: serum AIF1 concentrations correlate with HgbA1c and waist circumference in a Japanese population^2^, sequence variants near the *AIF1* locus are associated with adult obesity in Greeks^3^, and a recent report characterizes AIF1 as an adipokine secreted by macrophages in the white adipose tissue of obese women^4^. Furthermore, *in vitro* studies showed that conditioned media from the RAW 264.7 macrophage cell line overexpressing AIF1 induces pro-inflammatory genes (*Tnf-*α*, IL-6*), limits glucose uptake, and inhibits insulin signaling in differentiated 3T3L1 cells – a cellular model of white adipocytes^5^.

Despite strong structural similarity with AIF1^6^, AIF1L (also called IBA2) – first identified by the Human Genome Project – has not been studied in a metabolic context. The limited literature describing AIF1L function links it to proliferation and migration of breast cancer cells^7,8^ and regulation of actomyosin contractility and filopodial extensions in podocytes^9^. Expression of *Aif1l* has been described in kidney, heart, lung, and brain^7^, but has not been reported in adipose tissues.

AIF1L has a predicted molecular weight of 17 kDa, with amino acid sequence identity of 63% and similarity of 80% when compared to AIF1. The two proteins belong to the EF hand-bearing superfamily of proteins^10^, share similar molecular structures, and have indistinguishable F-actin bundling activity *in vitro*^6^. Despite the similarities between AIF1 and AIF1L, it remains unclear whether these proteins have cooperative, complementary, or redundant roles in mammalian physiology or pathophysiology. Because of the homology of these members of the AIF family and reported AIF1 associations with metabolism and obesity, we hypothesized that AIF1L might play a role in diet-induced obesity (DIO) and associated glucose insensitivity.

Obesity is among the most important public health challenges of modern times. In the U.S. alone, approximately 2 in 3 adults are overweight or obese, 1 in 3 is obese, and 1 in 20 is morbidly obese. Although traditional views of obesity pathogenesis focused on lifestyle, it is now well established that interactions between diet, environment, and genetic factors play critical roles in the susceptibility and severity of several metabolic diseases, including obesity. In addition, obesity is either associated with or poses an independent risk for the development of several pathophysiological conditions such as cancer^11^, cardiovascular diseases^12^, and non-alcoholic-fatty liver disease^13^.

Obesity is a major risk factor for insulin resistance (IR), which is closely linked to Type 2 diabetes in human subjects. Obesity has also been associated with chronic inflammation in visceral adipose tissue^14,15^, which is in turn associated with the development of IR. However, emerging evidence and independent studies from different groups show that IR can develop after only 3 days^16,17^ or 4 weeks of HFD feeding^18^, whereas an increase in pro-inflammatory macrophages is not seen until 10 weeks of HFD feeding^18^. Early development of IR is initiated as an acute response to HFD consumption, while long term HFD-induced IR is dependent on adipose tissue inflammation characterized by macrophage infiltration and accumulation^16^. In addition, IR itself can lead to additional monocyte/macrophage infiltration^18^; creating a vicious cycle. However, detailed molecular mechanisms defining these events are yet to be identified and understood. Cells of macrophage lineage display remarkable phenotypic plasticity in response to external stimuli^19^. AIF1 has been primarily characterized as a macrophage cytoplasmic protein that plays a role in phagocytosis, migration, and survival^20–23^, and it also supports the expression of inflammatory factors including IL-6, IL-12, and IFN-gamma *in vivo*^24^. Whether AIF1L also promotes inflammatory signaling or cytokine production is not known.

This is the first study to investigate to report the functional role of AIF1L in the context of metabolism and obesity. Here, we note that AIF1L is detected in the major adipose depots of mice fed with either normal diet (ND) or high fat diet (HFD). We also report that mice lacking AIF1L and fed HFD exhibit no differences in food intake, weight gain, fat mass, or energy expenditure compared to control mice. In addition, we found that loss of AIF1L did not change systemic glucose or insulin sensitivity in 8–9-week-old mice fed ND or HFD-induced glucose insensitivity.

## Results

### AIF1L is expressed in metabolic tissues of adult mice at baseline and upon HFD feeding

**Figure 1:**
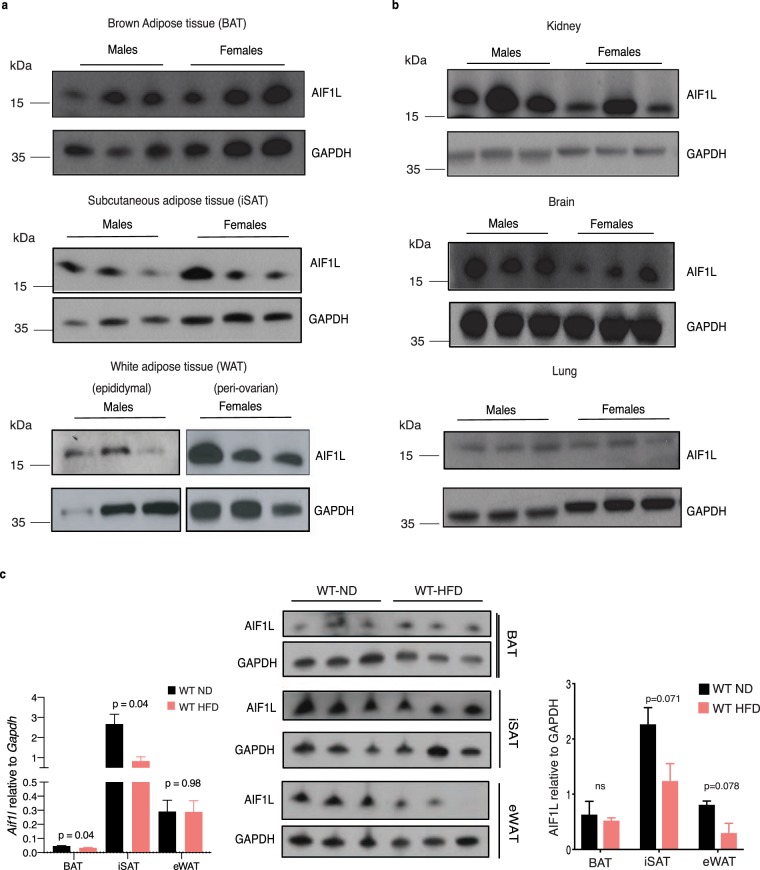
AIF1L is expressed in adipose depots, kidney, brain, and lung derived from adult male and female mice. (**a**) AIF1L protein expression in BAT, SAT and eWAT from 8-week-old adult male (n = 3) and female mice (n = 3). (**b**) AIF1L protein expression in kidney, brain, and lung. (**c**) Expression of *Aif1l* and AIF1L in adipose depots from age matched WT mice fed ND or HFD for 18 weeks, starting at 8 weeks of age (n = 3). Data are represented in mean ± SEM. ns – not significant.

AIF1 is expressed predominantly in testis and immune-associated organs such as spleen^25,26^, whereas the tissue distribution of AIF1L has not been established. RT-PCR analysis showed *Aif1l* transcripts in mouse kidney, heart, brain, lung, spleen, and skeletal muscle^7^, but mRNA expression in adipose tissue has not been reported to date. To permit AIF1L protein detection, we first assessed the specificity of the anti-AIF1L antibody relative to AIF1, using AIF1L-deficient (described below) and AIF1-deficient tissues as controls. Immunoblotting with anti-AIF1L antibody showed an appropriate 17 kDa band in kidney, but not in spleen (where AIF1 is highly expressed), while an anti- AIF1 antibody gave the opposite pattern **(**Supplementary Fig. S1**)**. This result indicates that this anti-AIF1L antibody can discriminate between AIF1 and AIF1L. In studies of ND-fed WT mice, we found AIF1L expression in all 3 adipose tissue depots – brown (BAT), inguinal subcutaneous (iSAT), and epididymal white adipose tissue (eWAT) **(**Fig. 1a**)**. In addition to this signal in adipose depots, immunoblotting showed a strong 17 kDa band in kidney **(**Fig. 1b**)**; this band was lacking in kidney lysates from AIF1L deficient mice(strategy described below and in methods section), confirming its identity as AIF1L **(**Supplementary Fig. S2**)**. In addition, we observed moderate levels of AIF1L expression in brain, and low levels in lung **(**Fig. 1b**)**, but no signal in liver or heart **(**Supplementary Fig. S2**)**. Skeletal muscle from WT mice showed bands of ~14 and 17 kDa in immunoblotting; because similar bands were observed in skeletal muscle from AIF1L deficient mice, these most likely represent cross-reaction with an unknown protein, as further discussed in the figure legend, and we believe that expression of AIF1L in skeletal muscle is very low compared to other organs we tested and undetectable with the methodology used here. Possible cross-reactivity of the anti-AIF1L antibody with AIF1, which also migrates at 17 kDa, was excluded because the same antibody did not cross react with AIF1 in spleen, as noted above (Supplementary Fig. S1) further indicating that the anti-AIF1L antibody is cross-reacting with a protein of ~17 kDa expressed selectively in skeletal muscle that is neither AIF1L nor AIF1. The identity of this cross-reacting protein is unknown. While we did not find any significant differences in AIF1L expression between males and females in BAT, iSAT, and brain, we observed a trend towards increased expression in WAT from females compared to males **(**Supplementary Fig. S2**)**. Considering the cellular heterogeneity of adipose tissues, we wanted to know if AIF1L is expressed in adipocytes, and used the well-known 3T3L1 cell line, an inducible *in vitro* model of white adipogenesis. We checked AIF1L expression at baseline, during differentiation, and after differentiation, and found that AIF1L is expressed in each of these stages. Interestingly, we could not detect AIF1L in the macrophage cell line, RAW 264.7, which is known to express AIF1^1^ **(**Supplementary Fig. S1). Thus, AIF1 and AIF1L show distinct patterns of expression among different cell types.

We further assessed if the expression of AIF1L is regulated in adipose depots upon HFD feeding. Tissues harvested from WT mice fed with either ND or HFD for 18 weeks were tested for *Aif1l* levels using qRT-PCR. We found expression in all three major adipose depots with the lowest in BAT and highest in iSAT from ND-fed WT mice. Upon HFD- feeding, we still found *Aif1l* expression in these tissues but at a lower level in BAT and iSAT compared to ND-fed WT mice. However, although we found no significant differences in AIF1L protein levels between the diet groups, a trend toward decreased expression was observed in iSAT and eWAT of the HFD group **(**Fig. 1c**)**.

### AIF1L-deficient mice are born in predicted Mendelian ratios, grow normally, breed well, and reach adulthood

AIF1L-deficient mice have not been previously described. We utilized mice bearing a modified *Aif1l* allele developed by the Wellcome Trust Sanger institute (WTSI)/Knockout Mouse Project (KOMP); this knockout (KO)-first strategy inserts a splice acceptor-*lacZ* cassette into intron 2 of the *Aif1l* gene **(**Supplementary Fig. S3). Mice heterozygous for this allele (*Aif1l*^*WT/lacZ-flxneo-flxex3*^) were mated to obtain WT and homozygous *Aif1l “*KO-first” mice. Further crosses with E2a-Cre transgenic mice^27^ induced Cre-mediated recombination in early zygotes to yield a global *Aif1l* locus allele with the splice acceptor-*lacZ* cassette but lacking *Aif1l* exon 3 and the neomycin selection cassette **(**Supplementary Fig. S3) (*Aif1l*^*lacZ/lacZ*^; referred as KO hereafter), in turn resulting in loss of AIF1L expression.

**Figure 2:**
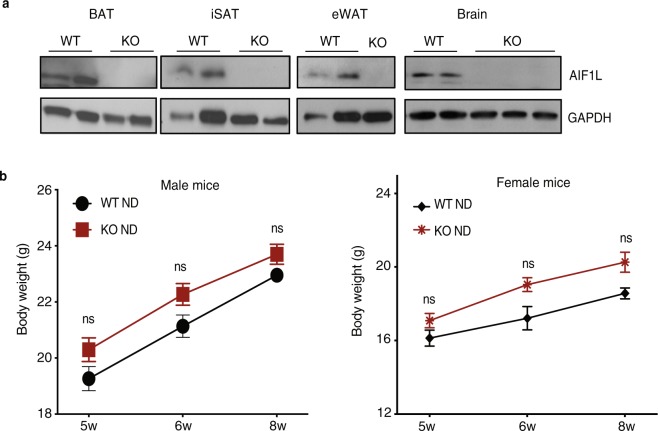
The modified Aif1l locus in mice results in loss of AIF1L expression and does not affect weight gain from 5 to 8 weeks of age. (**a**) Immunoblotting for AIF1L in adipose tissues from WT and KO mice (n = 2). (**b**) AIF1L-deficient male (WT n = 9, KO n = 7) and female (WT n = 3, KO n = 7) mice show similar weight and weight gain from 5 to 8 weeks of age when compared to WT mice on normal diet. Data are represented in mean ± SEM. ns – not significant.

Genotyping by PCR analysis of genomic DNA from mutant mice confirmed the presence of *lacZ*, absence of the wild-type (WT) allele, and presence of the modified *Aif1l* allele **(**Supplementary Fig. S3**)**. As predicted based on the gene targeting strategy, analysis by RT-PCR using primers from *Aif1l* exons 1 and 2 yielded the expected products in both WT and KO kidney, while analysis with primers spanning exons 1 and 5 detected transcripts in WT, but not KO, kidney **(**Supplementary Fig. S3**)**. Furthermore, we observed distinct immunohistochemical staining for AIF1L in tubular epithelial cells and podocytes in the glomerulus of WT mice, and lack thereof in the KO mice **(**Supplementary Fig. S3**)**. Protein lysates from adipose tissues and brain were tested by immunoblotting, which showed a predicted 17 kDa band in wild type animals and lack thereof in KO mice **(**Fig. 2a**)**. In expanded breeding, we found that KO mice were born in Mendelian ratios **(**Table 1**)**, reached adulthood, and did not show any obvious phenotypic differences when compared to WT controls after following for 52 weeks. Importantly, WT and KO mice of both sexes showed similar growth and weight gain patterns from 5 to 8 weeks of age when fed a ND **(**Fig. 2b**)**.

**Table 1:** Genotype frequency in the progeny of *Aif1l*^*WT/lacZ*^ crossed with *Aif1l*^*WT/lacZ*^.

Genotype	Expected #	Observed #	Expected %	Observed %
Wild type (*Aif1l*^*WT/WT*^)	30	38	25	31.67
Heterozygous (*Aif1l*^*WT/lacZ*^*)*	60	54	50	45
Homozygous (*Aif1l*^*lacZ/lacZ*^*)*	30	28	25	23.33
Total	120	120	100	100

No significant deviation observed from the expected mendelian ratios; Chi Square = 2.867 with 2 df, p = 0.2385.

To assess for possible compensatory regulation of AIF1 in AIF1L KO mice, we evaluated AIF1 expression levels in the key metabolic organs and spleen from adult mice (8–9 weeks old), using immunoblotting with lysates from bone marrow derived macrophages (BMDM) as controls. We found downregulation of AIF1 in BAT from AIF1L KO mice but a trend towards increased expression in eWAT **(**Supplementary Fig. S4). These contrasting patterns of AIF1L-dependent AIF1 regulation according to adipose tissue type are curious; the increase in AIF1 levels could reflect compensation in eWAT, but clearly a different mode of regulation is operating in BAT.

### Loss of AIF1L does not affect body weight or body composition upon short-term high fat diet (HFD) feeding

**Figure 3:**
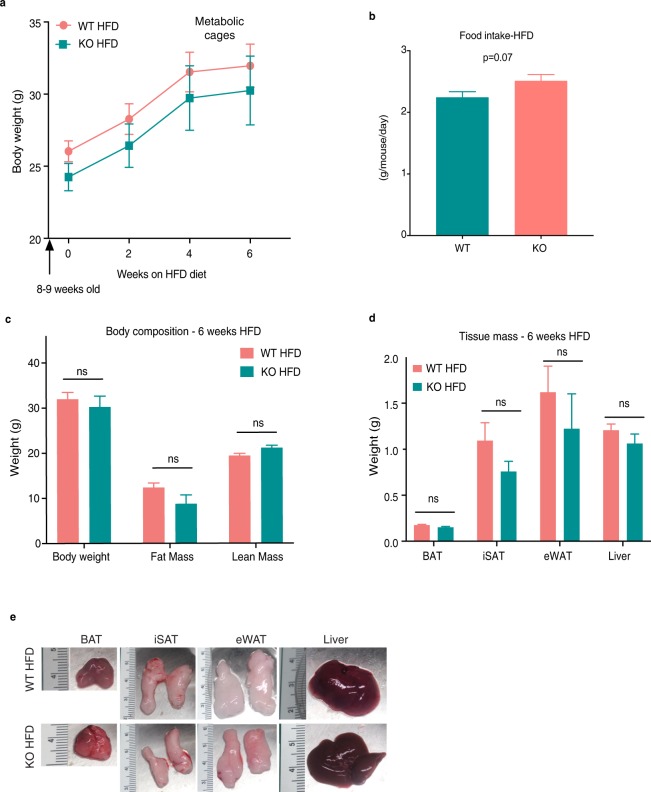
Loss of AIF1L does not affect total weight gain or body composition during short term HFD feeding. (n = 3 for WT and n = 4 for KO). (**a**) Weight gain over 6 weeks of HFD feeding (Flattening of the curves at week 6 in these mouse cohorts followed a move to the Animal Physiology core facility for subsequent metabolic cage and MRI studies; other cohorts (*e.g*., Fig. 4a, Supplementary Fig. 5a) used in weight gain or glucose sensitivity studies did not show similar flattening of the weight curve.). (**b**) Food intake over 5 weeks of HFD feeding (n = 3 for WT and n = 4 for KO). (**c**) Body weight, fat, and lean mass measurements determined by MRI. (**d**) Brown (BAT), inguinal subcutaneous (iSAT), epididymal (eWAT) white adipose depot, and liver mass. (**e**) Macroscopic appearance of tissues - representative images. Data are represented as mean ± SEM. ns – not significant.

The expression of AIF1L in adipose tissues in both ND-and HFD-fed mice, together with the above-noted associations of its paralog AIF1 with metabolism and obesity, supported our rationale to further evaluate a potential role for AIF1L in obesity and glucose handling. After finding that mice lacking AIF1L showed no differences in growth or weight gain on ND through 8 weeks of age, we subjected adult WT and KO male mice to HFD and followed them for 6 weeks as a short-term HFD stress. We found no differences in weight gain curves **(**Fig. 3a**)**, food intake **(**Fig. 3b**)** or body composition **(**Fig. 3c**)** between WT and AIF1L KO mice. Adipose depots (BAT, iSAT, eWAT) and liver were harvested from the KO animals at the end of the study (6 weeks HFD feeding) and did not show any differences in their mass **(**Fig. 3d) and macroscopic appearance **(**Fig. 3e**)**, when compared to the WT mice. These results indicate that AIF1L is not essential for increased adiposity and weight gain upon short-term HFD feeding.

### Loss of AIF1L does not affect age-dependent or HFD-induced body weight gain and body composition

**Figure 4:**
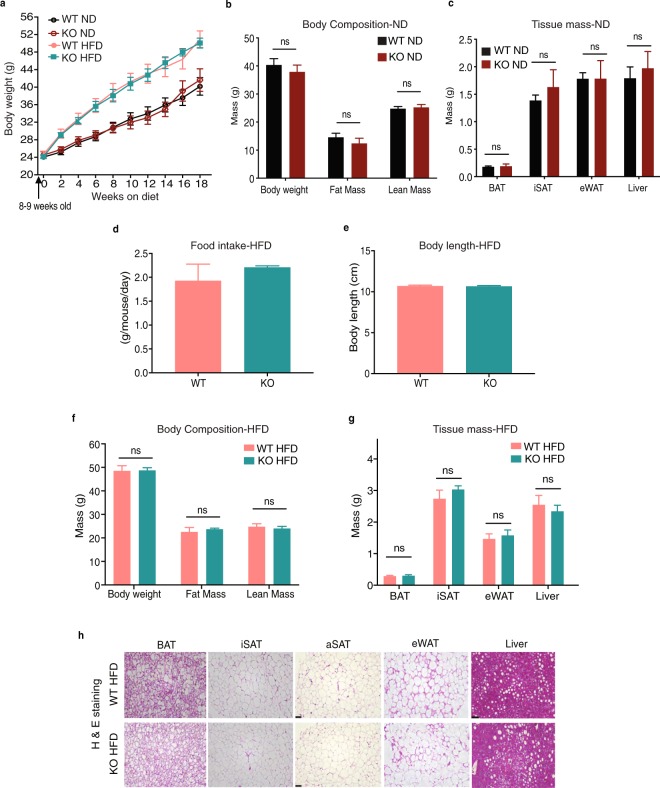
AIF1L does not affect total body weight or body composition during long term ND or HFD consumption. (**a**) Total body weight curves of WT (n = 6) and KO mice fed ND (n = 9) or HFD (n = 11 for WT, n = 8 for KO) starting at 8 weeks of age. **(b–c)** WT and KO mice on ND; age 26 weeks. (**b**) Body weight, fat, and lean mass measurements determined by MRI (n = 9 for WT, n = 8 for KO) (**c**) Brown (BAT), inguinal subcutaneous (iSAT), epididymal (eWAT) white adipose depot, and liver mass (n = 8 for WT, n = 3 for KO). **(d–h)** WT and KO mice fed with HFD for at least 18 weeks, starting at 8 weeks of age. (**d**) HFD consumption measured over 33 days (n = 6). (**e**) Body length (n = 3). (**f**) Body weight, fat mass and lean mass measured by MRI at the end of HFD feeding period for 18 weeks; age - 26 weeks. (n = 11 for WT, n = 8 for KO). (**g**) Brown (BAT), inguinal subcutaneous (iSAT), epididymal (eWAT) white adipose depot, and liver mass. (n = 11 for WT, n = 8 for KO). (**h**) H&E stained sections of adipose depot and liver sections - Representative images. (aSAT-anterior subcutaneous adipose depot). Data are represented as mean ± SEM. ns – not significant.

To evaluate the response to a longer metabolic stress, we either continued to follow the WT and KO mice on ND or switched to HFD feeding for 16–18 weeks, starting at 8–9 weeks of age. KO mice being followed on ND up to the age of 26 weeks displayed no change in weight gain curves **(**Fig. 4a**)** and body composition as measured by Echo-MRI compared to WT control mice **(**Fig. 4b**)**. No differences were observed between the 2 groups in their adipose depot and liver mass **(**Fig. 4c**)** or the macroscopic appearance of these organs **(**Supplementary Fig. S5**)**. These results show that AIF1L is not required for expected body weight increases on ND and age-dependent fat accumulation in adult mice.

On HFD, both WT and KO mice gained more weight than with ND, with no genotype-dependent differences in absolute body weight or weight gain over the period of HFD feeding **(**Fig. 4a**)**. WT and KO mice had similar food consumption, as measured over a period of 33 days **(**Fig. 4d**)**. The body length of the two groups was similar, suggesting normal overall growth of the animals **(**Fig. 4e**)**. We also performed micro-computed-tomography (μCT) scans, which showed no differences in fat accumulation in the visceral and subcutaneous adipose depots **(**Supplementary Fig. S5**)**, and Echo MRI studies, which showed no differences in fat and lean masses between the two groups **(**Fig. 4f**)**. Further analysis by harvesting and weighing the different adipose depots likewise revealed no differences in their masses and liver mass between WT and KO mice **(**Fig. 4g, Supplementary Fig. S5**)**. Histological differences could still contribute to overall similar mass of the depots and liver. In that regard, we wanted to check if the observed similar fat accumulation corresponds to similar adipocyte morphology within the adipose tissue between the two groups. Haematoxylin and Eosin (H & E) staining of tissue sections **(**Fig. 4h**)** revealed no obvious morphological differences. We further confirmed this by analyzing the size distribution of adipocytes in iSAT and eWAT sections and found no differences between the WT and KO HFD-fed mice. Furthermore, quantification of crown-like structures in these sections revealed no differences between the two groups, suggesting comparable degrees of infiltration of macrophages to the visceral adipose site **(**Supplementary Fig. S5**)**.

To further characterize the adipose tissue endocrine function, we checked gene expression of some of the major adipokines in iSAT and eWAT at baseline and from HFD-fed mice using quantitative PCR. These studies revealed genotype-dependent differences in adipokine expression: at baseline, *leptin, adiponection*, and *resistin* were lower in KO iSAT, consistent with lower serum levels of leptin found in KO animals, and this pattern of expression in adipose tissues was maintained with HFD **(**Supplementary Fig. S5**)**. In eWAT, on the other hand, adipokine transcripts were not different at baseline, but with HFD, these levels increased, with the largest relative increase noted for leptin. Whether the depot specific mRNA differences observed upon long-term-HFD feeding are translated to differences in serum levels will require further testing.

To uncover any possible sexual dimorphism role for AIF1L, we performed HFD feeding studies in female mice as well and measured the same parameters as mentioned above for male mice and found no differences **(**Supplementary Fig. S6**)**. Overall, despite these interesting effects on adipokine expression, these results combined suggest that AIF1L is not essential for the development of diet-induced obesity in male and female mice.

### Loss of AIF1L has modest effects on metabolic profile and activity in HFD-fed mice

**Figure 5:**
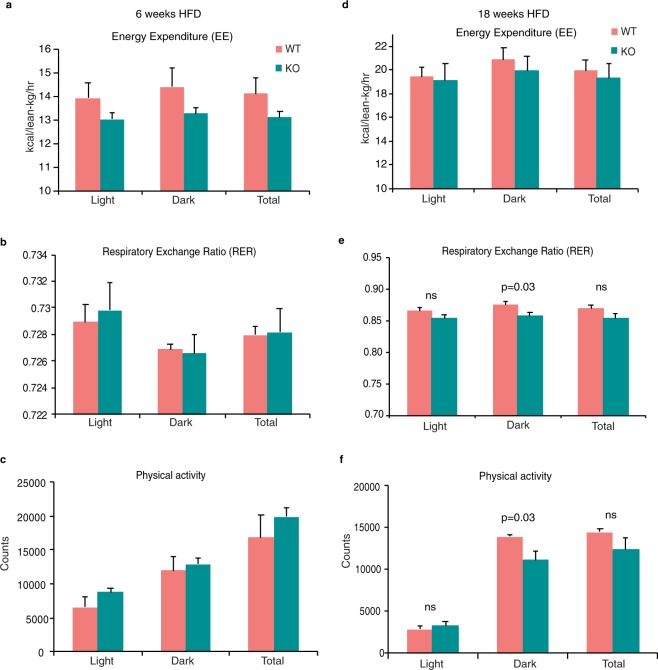
Loss of AIF1L does not alter metabolic profile with short- or long-term HFD feeding. **(a–c)** Metabolic cage studies performed on WT (n = 3) and KO (n = 4) mice fed HFD for 6 weeks (**a**) Energy expenditure (EE). Bar graph represents average EE values during day, night, and total. (**b**) Respiratory exchange ratio (RER; VCO2/VO2). Bar graph represents average RER values during day, night, and total. (**c**) Physical activity measured as beam breaks (Counts). Bar graph represents average beam break counts values during day, night, and total. **(d–f)** Metabolic cage studies performed on WT (n = 3) and KO (n = 3) mice fed HFD for 18 weeks. (**d**) Energy expenditure (EE). Bar graph represents average EE values during day, night, and total. (**e**) Respiratory exchange ratio (RER; VCO2/VO2). Bar graph represents average RER values during day, night, and total. (**f**) Physical activity measured as beam breaks (Counts). Bar graph represents average beam break counts values during day, night, and total. Data are represented in mean ± SEM. ns – not significant.

Since we did not observe differences between WT and AIF1L KO female mice upon HFD feeding, and that male mice have both higher sensitivity and lower variability in their susceptibility to metabolic syndrome^28^, we used male mice for the rest of the study. To determine if loss of AIF1L affects the overall metabolic profile of mice, we measured energy expenditure (EE), respiratory exchange ratio (RER), and physical activity of WT and KO mice after 6 or 18 weeks of HFD feeding. Measurement after 6 weeks of HFD feeding did not display any differences between WT and KO mice in their total EE, fuel source utilization, or physical activity either in the light or the dark cycle **(**Fig. 5a–c**)**. Measurements over a 24 h period are shown in Supplementary Fig. S7.

Even upon 18 weeks of HFD feeding, no significant differences were observed in EE between the 2 groups during the light or the dark cycle when monitored over a 24-hour period or average over 5 days of measurements **(**Fig. 5d, Supplementary Fig. S7**)**. An RER of 0.7 indicates that fatty acid oxidation is the main source of energy, whereas an RER of 1.0 or greater indicates that carbohydrates are the primary fuel source. Although RER values were reduced in KO animals and statistically significant during the dark cycle, when light and dark cycles are collectively analyzed, they were not different **(**Fig. 5e**)**. Similar observations were found for physical activity, showing different values between WT and KO mice during the dark cycle; with KO animals moving less; however, these differences disappeared when combined **(**Fig. 5f**)**. Twenty four-hour period measurements for RER and physical activity are shown in Supplementary Fig. S7. These results indicate that loss of AIF1L did not change total energy expenditure after a short course of HFD (6 weeks), but modestly suppressed dark period RER and physical activity after an extended course of HFD (18 weeks).

### Loss of AIF1L does not affect glucose handling at baseline or with HFD feeding

**Figure 6:**
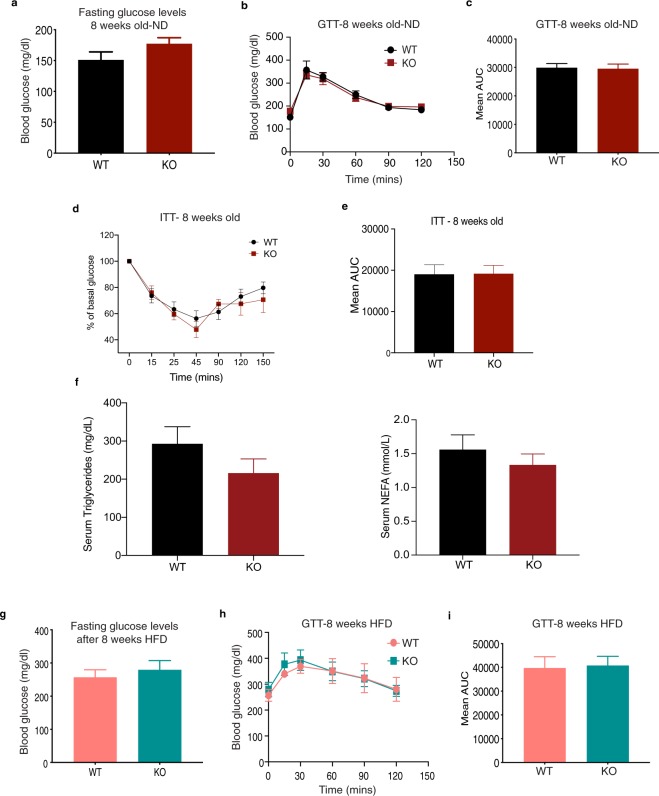
Loss of AIF1L does not affect glucose handling at baseline, or after short or long term HFD feeding. **(a–c)** 8-week-old adult male mice on ND. (**a**) Blood glucose levels in WT (n = 4) and KO (n = 4) mice after overnight fasting. (**b**) GTT with 1 g/kg (according to body weight) of glucose. (**c**) Quantification of ipGTT as mean area under curve (AUC). **(d–f)** 8–12-week-old adult male mice on ND. (**d**) Drop in glucose levels over time in WT (n = 7) and KO (n = 6) mice upon 0.75U/kg (according to body weight) of insulin challenge. (**e**) Quantification of ipITT as mean area under curve (AUC). (**f**) Serum triglycerides and NEFA levels (n = 3). **(g–i)** 8 weeks of HFD feeding. (**g**) Blood glucose levels in WT (n = 8) and KO (n = 7) mice after overnight fasting. (**h**) ipGTT with 0.5 g/kg (according to lean mass) of glucose. (**i**) Quantification of ipGTT as mean area under curve (AUC). Data are represented as mean ± SEM. ns – not significant.

Although obesity (BMI >30) is a major risk factor for the onset of glucose insensitivity, impaired glucose tolerance can also develop independent of the obese state or degree of obesity^29^. To test if this occurs with loss of AIF1L in mice, we first performed glucose tolerance test (GTT) at baseline – 8 weeks of age, and before starting the HFD feeding. No significant differences were observed between the WT and KO mice in their fasting glucose levels **(**Fig. 6a**)**, individual glucose measurements with GTT **(**Fig. 6b**)** and mean area under the curve (AUC) quantification **(**Fig. 6c**)**. These results indicate that AIF1L is not required to maintain basal glucose sensitivity. Furthermore, we found that there were no differences between the groups in their glucose handling upon insulin challenge **(**Fig. 6d,e**)** indicating similar sensitivity to insulin stimulated glucose clearance. We also checked serum levels of triglycerides (TGs) and non-esterified fatty acids (NEFA) in these mice and found no differences between the two groups **(**Fig. 6f**)**. These results combined suggest that AIF1L is not essential to maintain the overall metabolic health/key metabolic parameters in adult mice at baseline. We then checked if the HFD-induced stress could reveal a role for AIF1L in glucose handling. HFD feeding-induced glucose intolerance can manifest as early as 4 weeks after starting HFD consumption^18^. The same cohort of mice used for baseline GTT analysis were subjected to 8 weeks of HFD, after which significant increases in fasting blood glucose levels were observed in both groups compared to the ND-fed mice at age 8 weeks. However, the levels between the genotypes were similar **(**Fig. 6g**)**. GTTs between the HFD-fed groups were not different either in their individual glucose measurements **(**Fig. 6h**)** or AUC calculations **(**Fig. 6i**)**. These results show that both groups of mice are equally glucose intolerant. To differentiate the effect of age and HFD on glucose handling in mice receiving a HFD for 8 weeks, we performed GTT in 18-week-old male mice. Similar to our findings with mice raised on ND or fed a HFD for 8 weeks, we found no genotype-dependent differences between 18 week-old WT and AIF1L KO mice in terms of fasting blood glucose levels and AUC **(**Supplementary Fig. S8**)**. Thus, moderate aging did not reveal an effect of AIF1L on glucose handling.

## Discussion

In this study, we report that AIF1L expression in mice is detectable in the 3 major adipose depots – BAT, SAT, and eWAT – and also in kidney, lung, and brain. This corresponds to expression of AIF1L in human samples as reported in the Human Protein Atlas *(*https://www.proteinatlas.org/ENSG00000126878-AIF1L/tissue), thereby supporting the potential translational relevance of mouse models for understanding this little-studied gene. Using a genetic deletion in the mouse, we found that loss of AIF1L did not affect (1) embryonic development, (2) growth and maintenance of body weight from postnatal stage (5 weeks) to adult stage (8 weeks), (3) body composition or adiposity, (4) energy expenditure, or (5) glucose sensitivity. These findings indicate that AIF1L is not required for maintenance of chief metabolic parameters in mice. We demonstrate that loss of AIF1L does not affect age dependent weight gain and DIO up to age 26 weeks, nor does it modify basal glucose and insulin sensitivity or HFD-induced glucose insensitivity.

We observed a slight reduction in RER values for KO (0.85) mice during the dark period compared to WT mice (0.87), but these values are still within the range of the same substrate utilization as the WT mice. Interestingly, we found reduced physical activity levels during the dark period in AIF1L KO animals after long-term HFD feeding, despite similar body weight and energy expenditure. Given the robust expression of AIF1L in whole brain demonstrated here, and corresponding reports in human samples, these results suggest a possible role for AIF1L in the context of circadian biology bearing on activity. It should also be noted that this difference was only observed upon long term HFD feeding and not in the 6 week HFD-fed cohort; in view of the similar HFD-dependent body composition and metabolic functions among these groups, this difference in activity could reflect an AIF1L-dependent effect of aging rather than metabolism per se.

We also found reduced *leptin* mRNA expression in iSAT and reduced serum levels from 8–9-week-old AIF1L KO adult male mice when compared to the WT controls. Leptin is a critical regulator of appetite and energy expenditure^30,31^; while we did not observe any gross food intake differences in either sex when animals are group housed, the slightly lower physical activity observed during the dark phase in KO mice could be due to lower leptin levels in the KO animals. Because proper functioning of circadian rhythm itself regulates *leptin* transcription via Clock proteins^32^ and perturbations of such rhythms might be linked to the slight changes in dark-phase-specific physical activity in AIF1L KO animals, it is possible that these three observations are mechanistically linked, pointing to a need for further investigation of this potential novel regulator of leptin.

Loss of AIF1L did not affect glucose insensitivity in HFD-fed mice for 8 weeks. Although HFD-induced glucose intolerance could be seen as early as 4 weeks of HFD feeding^17,33^, effects of long-term (16–18 weeks) HFD feeding on glucose handling in these mice will require further testing. Considering that inflammation of the adipose organ is one of the key links between obesity and developing glucose intolerance^15,34,35^, we anticipate similarly impaired response between the control and AIF1L KO mice upon glucose challenge, based on the similar quantitation of crown like structures shown in Supplementary Fig. S5, one of the markers of inflammation.

Tremendous progress has been made in understanding adipose tissue function in the context of metabolic and its associated complications^36–38^. Despite this progress, how two major cell populations present in adipose tissue – adipocytes and macrophages – interact in an obese state is not fully understood, and this relationship is further complicated by sex-specific differences. Equally critical, the detailed molecular mechanisms by which macrophages in this inflammatory milieu endure and maintain their pro-inflammatory states have yet to be elucidated.

We hypothesized that the characterization of a novel candidate protein, AIF1L, in a mouse model of obesity could help to discover novel regulatory proteins acting in key pathways. Based on our study and published reports on AIF1, it appears that AIF1 and AIF1L proteins are both detectable in adipose tissues, but it is possible that they are expressed in different cell types of this heterogenous endocrine organ. We identified AIF1L expression in 3T3L1 cells under basal conditions and in differentiated white-adipocyte-like cells, but we could not detect it in mouse spleen or immortalized macrophages **(**Supplementary Fig. S1**)**. In contrast, AIF1 is well characterized in macrophages. Our results also indicate that AIF1 expression in brown and visceral adipose depots at baseline is differentially affected by global loss of AIF1L. Further systematic studies will be required to determine if this trend continues after switching to a HFD and whether this regulation of expression has pathophysiological significance. These differences in expression among distinct cell types within adipose tissues and divergent regulation in different adipose depots also advocate for further investigation of these proteins in adipose tissue biology. The current study provides new information regarding the expression and functional significance of one member of the AIF family. The corresponding role of AIF1L’s paralog – AIF1 – in the same context has yet to be reported.

The downregulation of AIF1L expression observed in the intermediate stage of 3T3L1 cell differentiation suggests that AIF1L might limit adipogenesis and/or lipogenesis. However, our *in vivo* studies did not show any differences in the overall body weight during the adolescent period – starting at 5 w of age until 8 w of age – as shown in Fig. 2b. Taken together, these observations suggest that factors not present in the 3T3L1 system may compensate for the germline loss of AIF1L in our model and effectively obscure any adipogenesis-related phenotype. A prime candidate for such compensation is AIF1, the paralog of AIF1L; interestingly, AIF1 expression is differentially affected in BAT and eWAT depots by the loss of AIF1L, as shown in Supplementary Fig. S4.

Indeed, the existing literature that addresses AIF1L function is very limited. Recently, AIF1L was shown to enhance cell proliferation via cyclin D1 in cellular models of breast cancer^7^, and to promote migration when overexpressed in cell lines^8^. In human subjects with cancer, higher levels of AIF1L correspond to higher tumor grade and worse prognosis^7,8^. On the other hand, AIF1L has also been shown to have a role in regulating actomyosin contractility and filopodial extensions, in turn maintaining human podocyte morphology and integrity^9^.

At the protein level, AIF1L and AIF1 show strong conservation of amino acid identity as well as structure^6^. AIF1 has been extensively characterized as a macrophage cytoplasmic protein that functions in phagocytosis, migration, survival, and serves as a marker of activation in microglia^39^. This robust expression of AIF1 in macrophages has been linked to pathophysiological conditions such as rheumatoid arthritis (RA)^40,41^ in human subjects. Mouse models and *in vitro* studies display a significant role for AIF1 in autoimmune diseases such as experimental autoimmune encephalomyelitis (EAE), animal model of multiple sclerosis^24,42^, neuritis^42,43^, and type 1 diabetes in non-obese diabetic mice^44^. In addition, like AIF1L, AIF1 has also been reported to be upregulated in breast ductal tumor epithelia compared to normal epithelia^45^, and to promote cell proliferation^45^ and migration in breast cancer cell lines^46^. At the molecular level, the only known common function of these paralogs is their actin bundling activity^6^. These functional observations and disease associations for AIF1, along with the structural similarities of AIF1 and AIF1L led us to explore functions of the latter protein.

A possible limitation of our study is the use of mice with global loss of AIF1L throughout development, which could result in compensation by factors that mask a phenotype. Furthermore, our expression data for AIF1L and differences in leptin expression with AIF1L deficiency suggest a role in adipose tissue biology; spatiotemporally regulated loss of AIF1L could circumvent the problem of compensation and thereby reveal such a role. It is also difficult to fully exclude subtle phenotypes that might be revealed by either tissue specific deletion or by exposure to different metabolic stresses.

In conclusion, we have found that AIF1L is expressed in major metabolic tissues, but is not essential for survival, grossly normal development, or reproduction. In focused studies of AIF1L function in models of metabolic stress, including a HFD model of obesity, we found that loss of AIF1L did not affect maintenance of age-dependent body weight, HFD-induced weight gain, body composition, adipose depot size, or obesity-induced impairment of glucose handling. These findings show that while AIF1L is not required for the pathogenesis of diet induced obesity and glucose insensitivity, its expression pattern in adipose tissues and brain and regulation of AIF1 expression in AIF1L KO mice suggest that this protein family may yet have a regulatory role in this context, and use of temporally-controlled or tissue specific KO models may uncover these anticipated functions.

## Materials and Methods

### Mice

Mice bearing the *Aif1L* “KO-first” allele on a C57BL6/N background were obtained from Knockout mouse project repository (KOMP). They were backcrossed for 8 generations to the C57BL6/J strain before mating them with E2a-Cre transgenic mice (Jackson laboratories #003724) to generate AIF1L-deficient mice with the *lacZ* gene driven by the endogenous *Aif1l* promoter. All animals were housed in pathogen-free conditions, and procedures were in accordance with the rules and regulations of the Association for Assessment and Accreditation of Laboratory Animal Care (AAALAC) and were approved by the Institutional Animal Care and Use Committee (IACUC) of the Albert Einstein College of Medicine. At least two of the cohorts used were littermates. Breeders of non- littermate cohorts came from the same founder animals. One of the three cohorts used in the weight gain studies came from breeding *Aif1l*^*WT/lacZ*^*; E2a-Cre*^*Tg*^ with *Aif1l*^*WT/lacZ*^ and rest of them came from either *Aif1l*^*WT/lacZ*^ with *Aif1l*^*WT/lacZ*^ or *Aif1l*^*WT/WT*^ with *Aif1l*^*WT/WT*^ or *Aif1l*^*lacZ/lacZ*^ with *Aif1l*^*lacZ/lacZ*^.

### Genotyping

Genotyping was done using genomic DNA extracted from tail tips using tail lysis buffer and proteinase K with overnight digestion at 56 °C. Proteinase K was deactivated at 92 °C for 20 minutes and samples were centrifuged to remove the debris. Supernatant was used to perform PCR reactions. Primers used for genotyping are mentioned in Table 2. To confirm if the recombination occurred in the mice bearing the E2a-Cre transgene, we designed primers spanning the intron 2-exon 3 junction.

**Table 2:** Primers used.

Primer name	Primer Sequence 5′ to 3′	Position on modified *AIF1L* allele
**For genotyping**		
Aif1l_39300_F	GCTCCTGCACTCTGCATCTC	14699–14718 (From WTSI*)
Aif1l_39300_R	ACCTTCCCCACTCTGTGCTC	22236–22255 (From WTSI)
CAS_R1_Term	TCGTGGTATCGTTATGCGCC	15053–15072 (From WTSI)
LacZ_2_small_F	ATCACGACGCGCTGTATC	18203–18220
LacZ_2_small_R	ACATCGGGCAAATAATATCG	18291–18310
Exon3 forward	GATATCTCTATAGTCGCAGTAGGC	22147–22161
Exon3 reverse	CTTTGAAGGCTGCGAGCTTTTCC	22621–22643
**For cDNA analysis**	**Primer Sequence 5′ to 3′**	
Exon1_F	ATGTCGGTCGCACTCAGCAA	
Exon5_R	CTGACACCCCCTGTCACCTC	
Exon2_R	CCGATTGATCTCCTCGAGCCTC	
Adiponectin_F	GGTCTTCTTGGTCCTAAGGGTGAG	
Adiponectin_R	GCGGCTTCTCCAGGCTCTC	
Resistin_F	GCTGCTCCTGTGGCTCTGC	
Resistin_R	GGCTGCTGTCCAGTCTATCCTTG	
Leptin_F	AGGGAGGAAAATGTGCTGGAGAC	
Leptin_R	GATACCGACTGCGTGTGTGAAATG	

*WTSI- Wellcome trust Sanger Institute.

### RT-PCR

Total RNA was isolated from frozen tissues using Trizol reagent (Invitrogen; 15596018) according to manufacturer’s instructions. The RNA concentration was calculated using NanoDrop technology (ThermoFisher Scientific; ND-1000) and reverse transcribed to cDNA using Superscript III first strand synthesis system (Invitrogen 18080-051). Primers that were used to perform PCR are mentioned in Table 2.

Recombination of the modified *Aif1l* locus with Cre recombinase disrupts splicing after exon 2, leaving the *lacZ* cassette, and deletes exon 3. Exon 3 encodes aa 32–53 of the predicted AIF1L protein sequence (NP_660126). This overlaps the start of the putative EF hand motif in AIF1L at aa 47–82, so loss of exon 3 after Cre-mediated recombination would likely significantly affect protein structure and function. In addition, in the rare event of a read-through transcript resulting from a skipped splice acceptor in the *lacZ* cassette, deletion of exon 3 is expected to cause a frame shift after exon 2 in all possible transcripts that include exons 4, 5, and 6, resulting in a premature stop codon. These transcripts would most likely be degraded by nonsense mediated decay (NMD). Finally, in theory it is possible that translation might arise from an alternative start site downstream of exon 3 and result in peptides or truncated proteins with an intact AIF1L C terminus. Such products should be detectable by the antibody we have used, but IHC staining in kidney sections from AIF1L KO mice do not show any signal with this antibody. To confirm the loss of *Aif1l* mRNA transcript, we designed 2 sets of primers (1) spanning exon1 and exon 2, and (2) spanning exon 1 and exon 5. With this, we expected to see an amplicon resulting from exon 1 and exon 2 in both WT and KO mice, as shown in the Supplementary Fig. 3. With Set 2 we confirmed the loss of full length *Aif1l* transcript, as demonstrated by the presence of the expected amplicon in WT mice and lack thereof in the KO mice.

### Quantitative RT-PCR

RNA and cDNA were prepared as mentioned above. 10 μl reactions with 50 ng of cDNA were set up in triplicates using SYBR green (Applied biosystems, 4309155) and the assay was run in ViiA7 Real time PCR system (Applied biosystems). The data was analyzed with 2^−ΔCt^ method in which ΔCt was calculated between the gene of interest and housekeeping gene, *18S rRNA* or *Gapdh*, as indicated.

### Immunoblotting

Frozen tissues were homogenized in RIPA lysis buffer (50 mM Tris-HCL pH 7.4, 1% NP40, 0.5% sodium deoxycholate, 0.1% SDS, 1 mM EDTA, 150 mM NaCl) with protease inhibitors (Complete, Roche) and phosphatase inhibitors (Roche; PhosSTOP Easypack 04906837001) for protein extraction and then centrifuged to defat and remove debris. Protein concentrations were measured by a BCA protein assay kit (Pierce). Equal amounts of protein (20–60 µg) were loaded, separated by 10–20% Tricine gel (Invitrogen; EC6625BOX) electrophoresis and blotted onto 0.2 μm pore size PVDF membranes (Immobilon-P, Millipore). After blocking in TBST (Tris pH 8.0, NaCl 150 mM, 0.1% Tween20) plus 5% (w/v) non-fat milk or BSA, blots were incubated at 4 °C overnight with primary antibodies. Signals were detected with horseradish peroxidase- conjugated secondary antibody and chemiluminescence (ECL blotting substrate, Pierce; 32106). Equivalent protein loading was tested with anti-GAPDH antibody (Santa Cruz, sc-25778, 1:5000 dilution). Primary antibodies used: anti-AIF1L (Atlas antibodies, HPA020522, 1:500 dilution), anti-AIF1 (Wako FUJIFILM, 016–20001, Abcam ab178847). Densitometric analysis was performed using ImageJ software.

### DIO models

Three different cohorts of animals were used for the studies. For one of the cohorts, mice were monitored and weighed at 5, 6, and 8 weeks of age before starting the HFD studies. At 8 weeks of age, the mice were either continued on ND (PicoLab® Mouse Diet 20 5058) or switched to HFD (Research Diets, D12492i; 60% of kCal from fat) for 18 weeks. The mice were weighed before switching and thereafter every 2 weeks. At the end of the feeding period, mice with a final weight of either 2 SD below or above the mean were excluded (n = 1 for each genotype) from the analysis.

### Food intake

A defined amount (2 g/day/mouse) of HFD food pellets were provided *ad libitum* to mice housed in groups of 3–5, and the amount remaining was measured on the third day. The amount consumed was averaged by the number of mice in the cage. This was done over a period of 33 days, with measurements every alternate day, starting at the first day of HFD feeding.

### Body composition and imaging

Lean mass and fat mass were acquired using an EchoMRI-100 Body composition analyzer. µCT scan was performed with the following settings – 0.08 mm, 30 slices, and slow speed.

### Indirect calorimetry analysis

Columbus Labs comprehensive lab animal monitoring systems (CLAMS) (Animal Physiology Core at Einstein) was used to collect multiparameter metabolic data, including energy expenditure (EE), physical activity as horizontal and vertical beam breaks, O_2_ consumption, and CO_2_ production to calculate respiratory exchange ratio (RER). All parameters were normalized to lean mass of each mouse. Data was continuously collected for 5 days after 48 h of acclimatization in the cages where mice were individually housed. These assays were done after either 5 weeks or 17 weeks of HFD feeding.

### Glucose sensitivity

Fasting blood glucose levels and intraperitoneal GTT were measured in tail blood using a OneTouch Ultra 2 glucometer, after fasting the animals overnight. Details of the assay are mentioned in figure legends.

### Insulin sensitivity

Adult WT and AIF1L KO mice (8–12 weeks old) were fasted for 10hrs before performing the assay. Fasting glucose levels were noted for both groups before challenging them with an ip injection of 0.75U/kg of insulin (Eli Lilly) according to body weight. Blood glucose levels were measured in tail blood at time points as labeled in the figure.

### Serum analysis

Serum from 8–12 weeks old adult mice on normal diet was collected without fasting using standard protocol and stored at −80C until analysis. Triglycerides (TGs) and Non-esterified fatty acids (NEFA) were measured colorimetrically with Beckman Coulter AU480 using Beckman Coulter Triglycerides reagent (OSR60118) & Fuji Film HR Series NEFA-HR solutions. Leptin levels were measured by ELISA according to manufacturer’s instructions (EZML-82K Milipore).

### Tissue preparation

Mice (8–9 weeks old) were anesthetized with an ip injection of ketamine and xylazine. Organs were isolated and a small piece from each was immediately frozen for RNA and protein isolation. Remaining parts were fixed in 10% formalin for 24 h and then transferred to 70% ethanol.

### Histological analysis

Tissue samples fixed for 24 h were processed and embedded in paraffin at the Einstein Histopathology core facility. They were sectioned at 5 µm and a routine H&E staining was performed. Adipocyte size measurements were performed using Adiposoft plugin for ImageJ with manual supervision and frequency distribution of the cell size was graphed using GraphPad prism. Crown like structures in eWAT were calculated manually using at least 3 fields per mouse.

### Statistics

Based on results of a pilot study for body weight gain curves on 60% HFD-fed WT mice, we determined the minimum number of mice per group that would be adequate to ensure 80% power to detect 75% change in mean weight gain, assuming a two-sided test and alpha of 0.05. For single time-point measurement, statistical analyses were performed using unpaired t tests with Welch’s correction for two groups to compare each group with WT mice unless otherwise specified. For repeated measurements, analysis of variance (ANOVA) was performed, with Sidak’s correction tests. AUC was measured for glucose and insulin tolerance tests. Chi square test was done to test statistical differences between observed and expected Mendelian ratios. Statistical analysis for frequency distribution curves were done with nonlinear regression fit gaussian model. All analysis was performed using GraphPad Prism 7 and data were expressed as mean ± SEM.

## Supplementary information

Supplementary information.

## Data Availability

Data presented here are available upon request, from the corresponding author.
