# Supplementary material for: Allograft inflammatory factor-1-like is not essential for age dependent weight gain or HFD-induced obesity and glucose insensitivity
Source: Sci Rep. 2020 Feb 27;10:3594. doi: 10.1038/s41598-020-60433-4 (PMC7046694; doi:10.1038/s41598-020-60433-4)
Supplement: Supplementary file 1 — Supplementary information. [file 41598_2020_60433_MOESM1_ESM.pdf]

Allograft inflammatory factor-1-like is not essential for age dependent weight gain or HFD-induced obesity and glucose insensitivity

Dippal Parikh[1], Dario F. Riascos-Bernal[1], Lander Egaña-Gorroño[1#], Smitha Jayakumar[1], Vanessa Almonte[1], Prameladevi Chinnasamy[1], Nicholas E. S. Sibinga[1*]

[1]Albert Einstein College of Medicine, Wilf Family Cardiovascular Research Institute, Department of Medicine (Cardiology), and Department of Developmental and Molecular Biology. 1300 Morris Park Avenue, Bronx, New York, 10461, USA.

[#] Present address: Diabetes Research Program, Division of Endocrinology, Diabetes and Metabolism, Department of Medicine, NYU Langone Medical Center, New York, NY 10016, USA.

Correspondence to Nicholas Sibinga, Albert Einstein College of Medicine, Forchheimer G46, 1300 Morris Park Avenue, Bronx, New York 10461, USA; phone 718-430-2881; fax 718-430-8989; e-mail: nicholas.sibinga@einstein.yu.edu

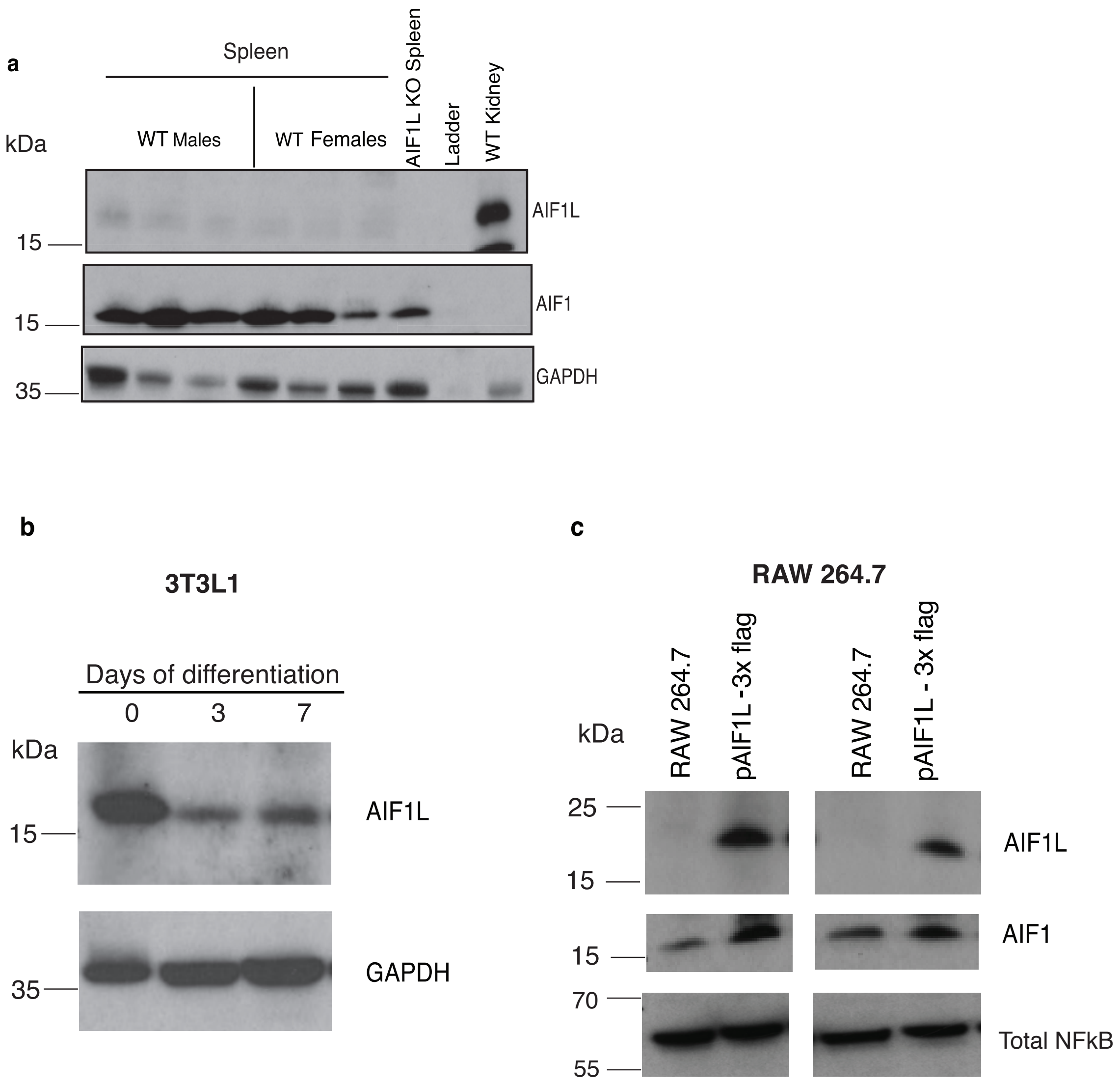

Supplementary Figure 1. Characterization of AIF1L antibody and its expression in cellular model of adipocytes

a) Immunoblot for AIF1L and AIF1 in spleen and kidney lysates from WT adult mice with anti AIF1L antibody (40µg of total protein loaded)

b) Immunoblot for AIF1L in 3T3L1 cells during induced adipogenic differentiation with anti AIF1L antibody (30µg of total protein loaded)

c) Immunoblot for FLAG-tagged AIF1L and endogenous AIF1 in RAW 264.7 cells with anti AIF1L and anti AIF1 antibodies respectively

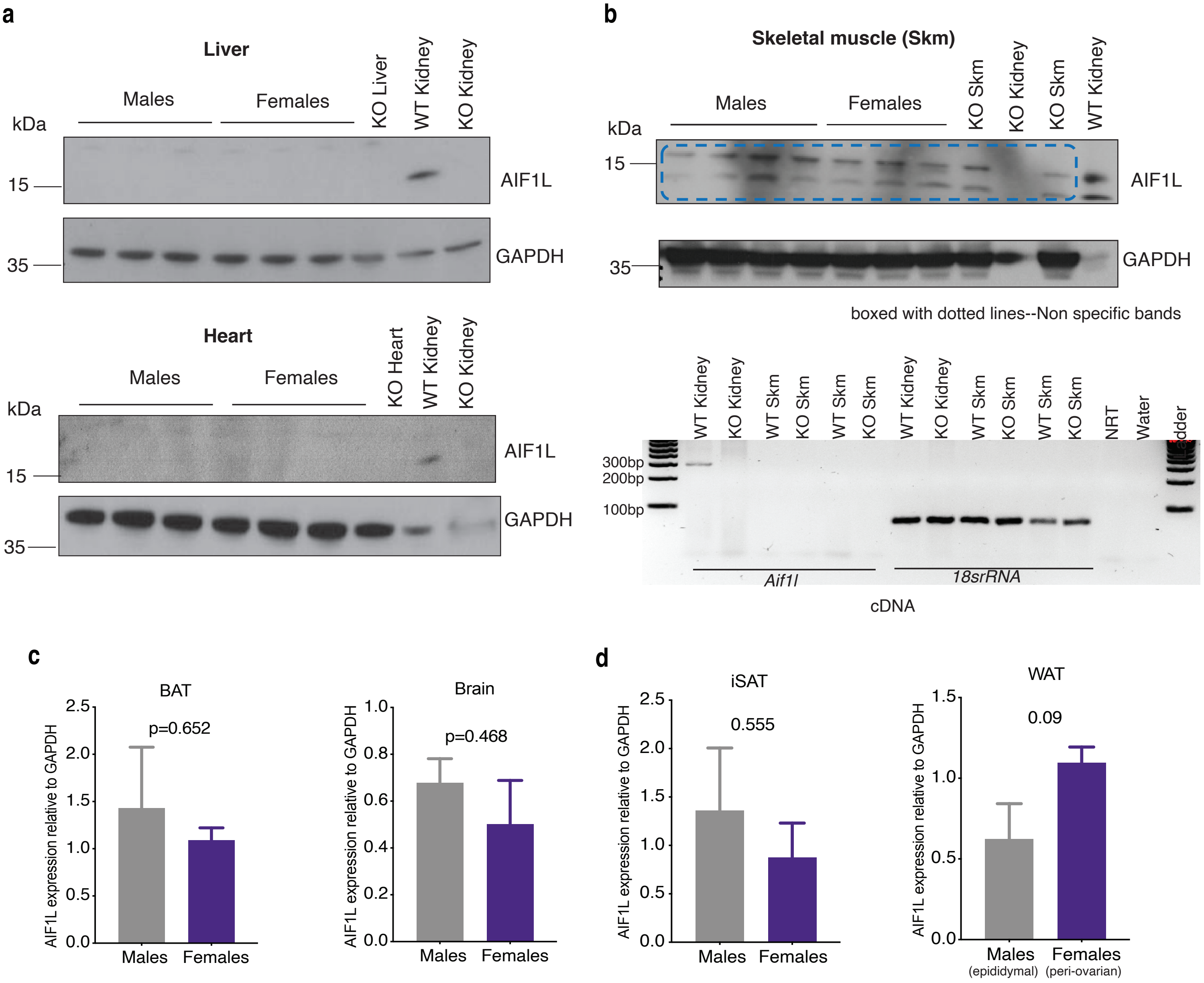

Supplementary Figure 2. Characterization of AIF1L expression in mouse tissues

a) Undetectable AIF1L expression in liver and heart
b) Undetectable AIF1L protein and mRNA expression in skeletal muscle (immunoblotting for AIF1L using skeletal muscle lysates from both WT and AIF1L KO mice showed bands of ~14 and 17 kDa that are most likely non-specific. The global KO strategy used in this case is effective, as confirmed by the loss of signal for AIF1L in kidney, where the protein is highly expressed in WT animals — suggesting that these bands in the skeletal muscle samples are not due to AIF1L. RT-PCR analysis of skeletal muscle RNA using primers spanning exons 1 and 5 yielded the expected ~300 bp band in WT samples, but no products with KO, including potential transcripts that might arise from exon skipping events; in any case, such events in the recombined *Aif1L* allele would be expected to yield smaller products (as discussed in Methods), and would not explain the 17 kDa band.)
c) Quantification of AIF1L expression in BAT and Brain
d) Quantification of AIF1L expression in white adipose tissues

Data are represented as mean ± SEM. ns - not significant.

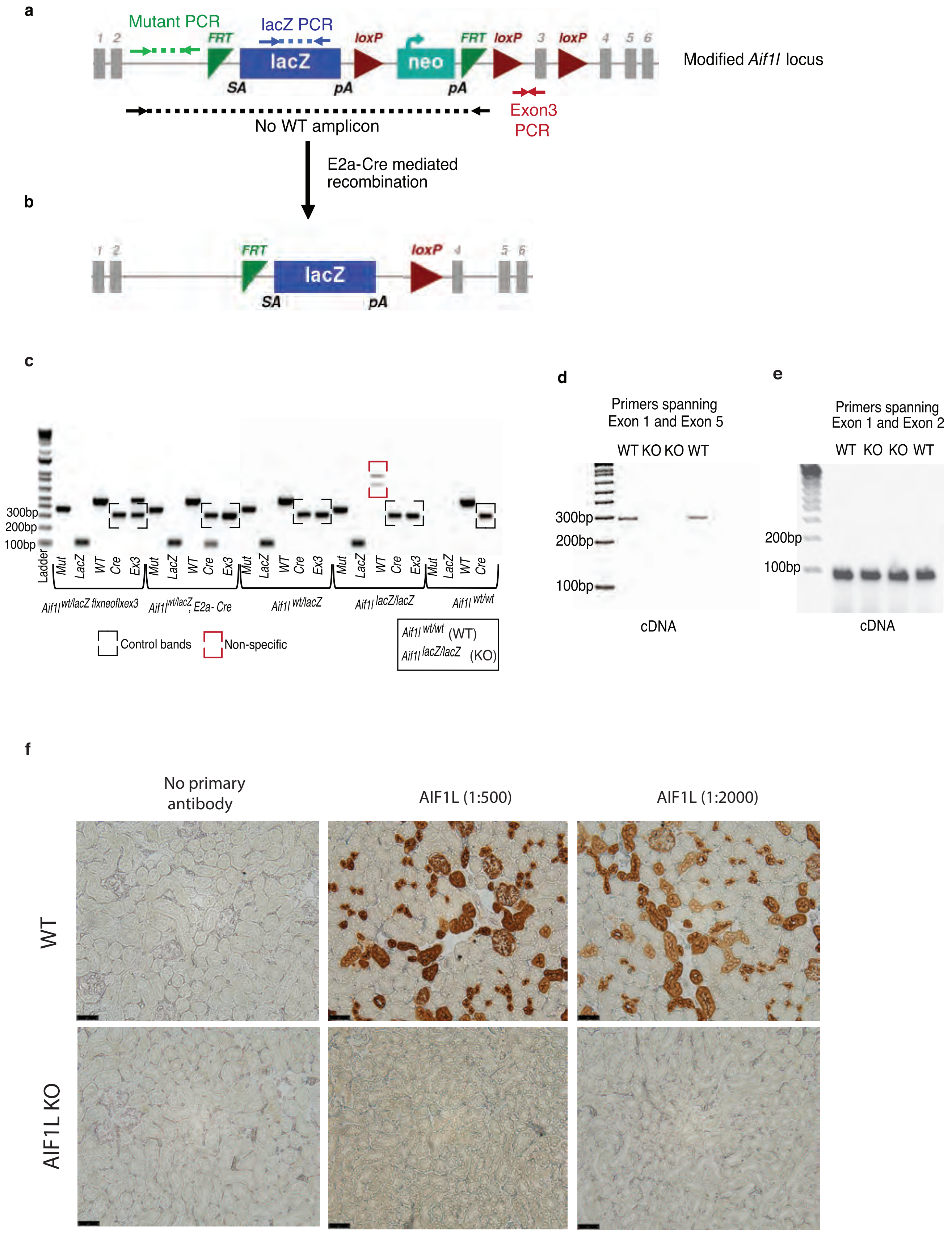
a
Mutant PCR
lacZ PCR
FRT
lacZ
loxP
neo
FRT
loxP
loxP
SA
pA
pA
Exon3
PCR
No WT amplicon
Modified Aif1l locus
E2a-Cre mediated
recombination
b
FRT
lacZ
loxP
SA
pA
c
300bp
200bp
100bp
Ladder
Mut
LacZ
WT
Cre
Ex3
Aif1l wt/lacZ flxneoflxex3
Aif1l wt/lacZ; E2a- Cre
Aif1l wt/lacZ
Aif1l lacZ/lacZ
Aif1l wt/wt
Control bands
Non-specific
Aif1l wt/wt (WT)
Aif1l lacZ/lacZ (KO)
d
Primers spanning
Exon 1 and Exon 5
WT KO KO WT
300bp
200bp
100bp
cDNA
e
Primers spanning
Exon 1 and Exon 2
WT KO KO WT
200bp
100bp
cDNA
f
No primary
antibody
AIF1L (1:500)
AIF1L (1:2000)
WT
AIF1L KO

Supplementary Figure 3 Modified *Aif1l* locus, tail DNA genotyping, and validation of loss of full length AIF1L

a) As described by Wellcome Trust Sanger Institute (WTSI) -- The L1L2_Bact_P cassette was inserted at position 31961760 of Chromosome 2 upstream of the critical exon(s) (Build GRCm38). The cassette is composed of an FRT site followed by *lacZ* sequence and a *loxP* site. This first *loxP* site is followed by a neomycin resistance gene under the control of the human beta-actin promoter, SV40 polyadenylation cassette, a second FRT site and a second *loxP* site. A third *loxP* site is inserted downstream of the targeted exon(s) at position 31962560. The critical exon is thus flanked by *loxP* sites.
b) Cre mediated recombination removes the neomycin cassette and exon 3, leaving one FRT site and *lacZ,* which is transcribed under the endogenous promoter of *Aif1l.*
c) Tail DNA genotyping from mice WT, heterozygous, or homozygous for the modified *Aif1l* allele.
d) End point PCR of *Aif1l* cDNA from WT (n = 2) and KO kidney (n = 2) with forward and reverse primers spanning exon 1 and exon 5, respectively.
e) End point PCR of *Aif1l* cDNA from WT (n = 2) and KO kidney (n = 2) with forward and reverse primers spanning exon 1 and exon 2, respectively.
f) Immunohistochemical (IHC) staining for AIF1L expression in kidney sections from 8-9-week-old WT and AIF1L KO male mice.

Abbreviations for genotyping PCR- Mut- presence of modified allele, *lacZ*- presence of *lacZ* cassette, Cre-presence of Cre, WT-presence of Wild-type allele, Ex3- presence of exon 3 in the modified allele.

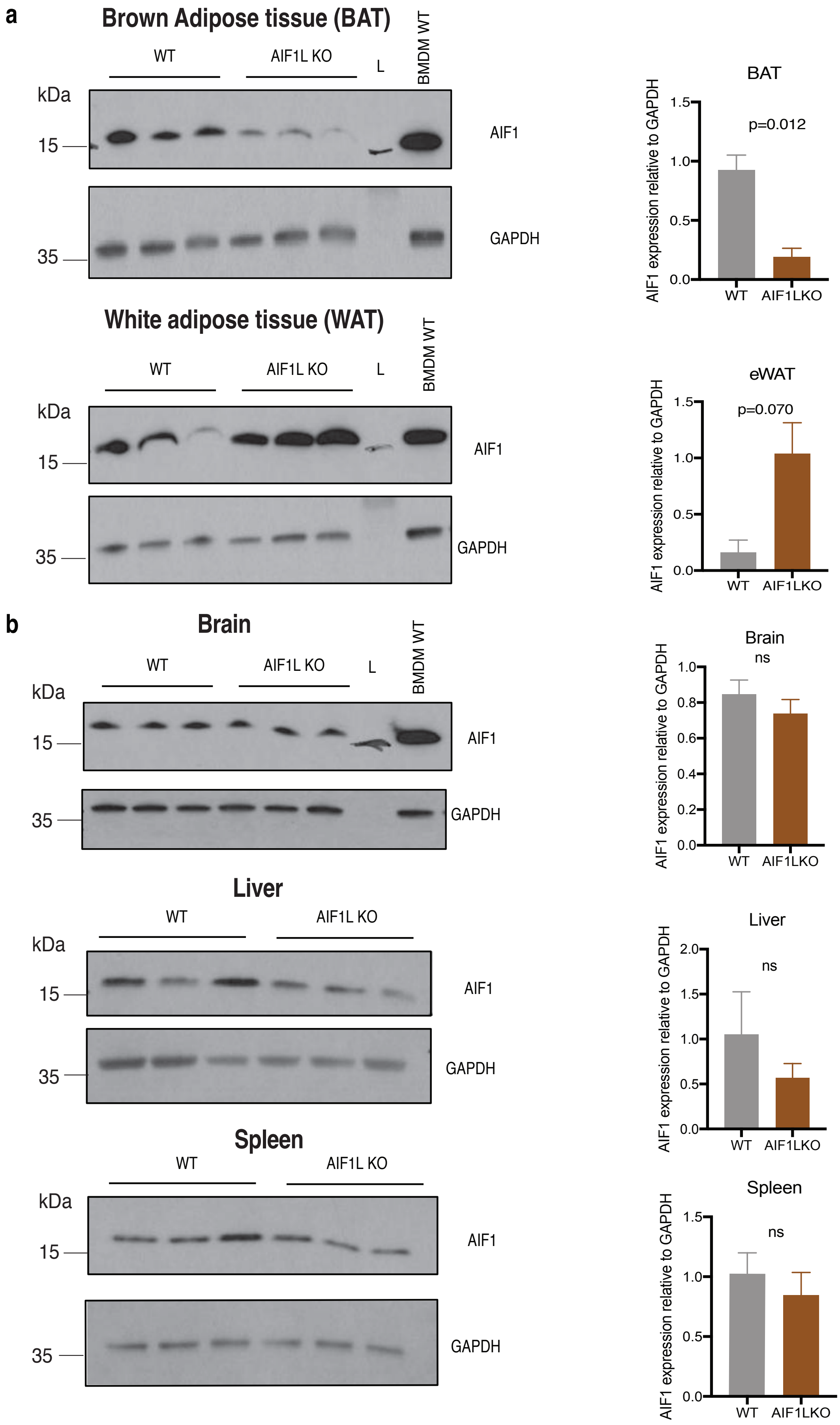

Supplementary Figure 4. AIF1 expression in tissues from WT and AIF1L KO 8-10 week old male mice

a) AIF1 expression in brown and epidiymal white adipose depots (n=3)
b) AIF1 expression in brain, liver, and spleen (n=3)

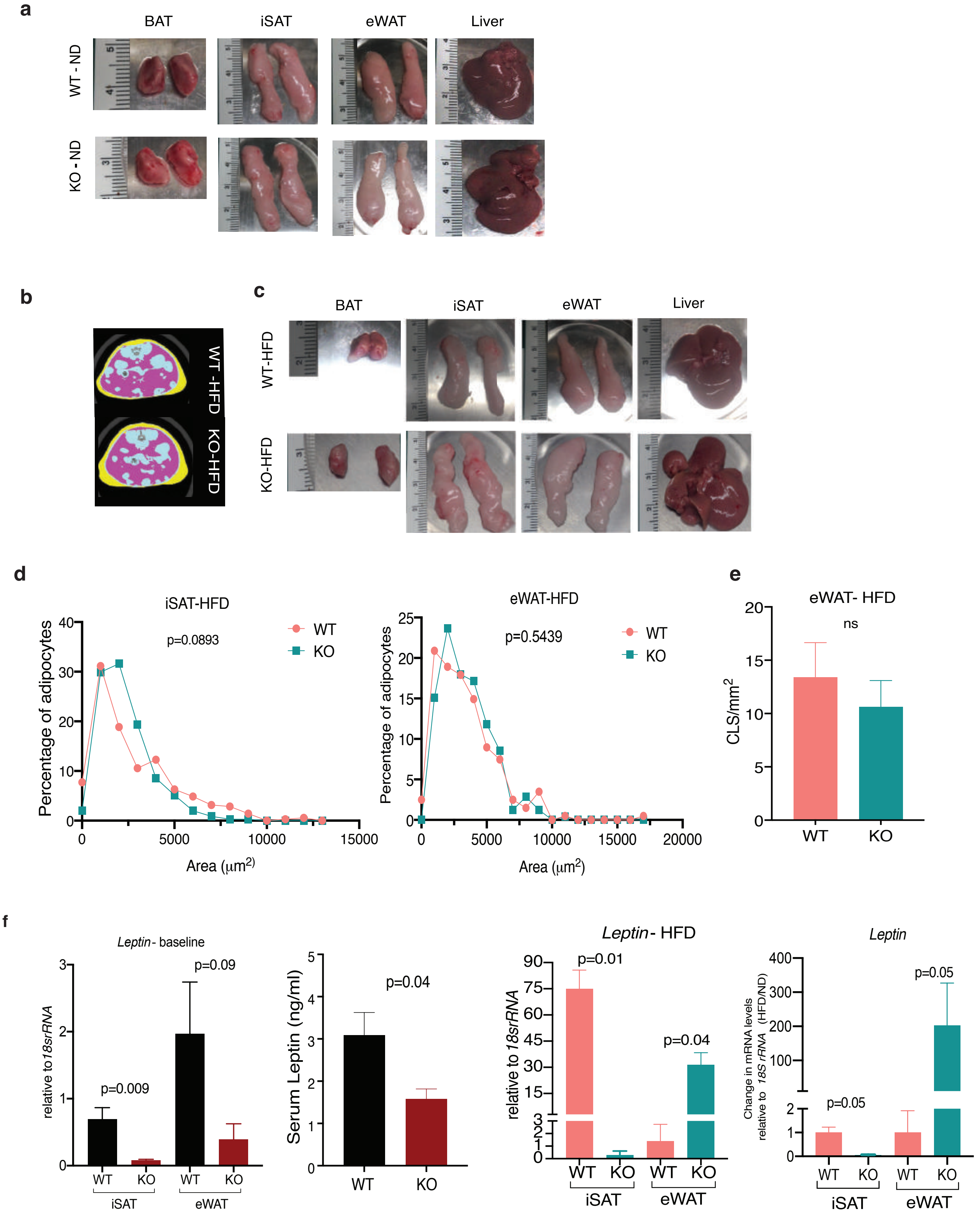

a
BAT
iSAT
eWAT
Liver
WT - ND
KO - ND
b
WT -HFD
KO-HFD
c
BAT
iSAT
eWAT
Liver
WT-HFD
KO-HFD
d
iSAT-HFD
p=0.0893
WT
KO
Percentage of adipocytes
Area (μm2)
eWAT-HFD
p=0.5439
e
eWAT- HFD
ns
CLS/mm2
WT
KO
f
Leptin- baseline
relative to 18srRNA
p=0.009
p=0.09
iSAT
eWAT
Serum Leptin (ng/ml)
p=0.04
Leptin- HFD
p=0.01
p=0.04
Leptin
Change in mRNA levels relative to 18S rRNA (HFD/ND)
p=0.05
p=0.05

g

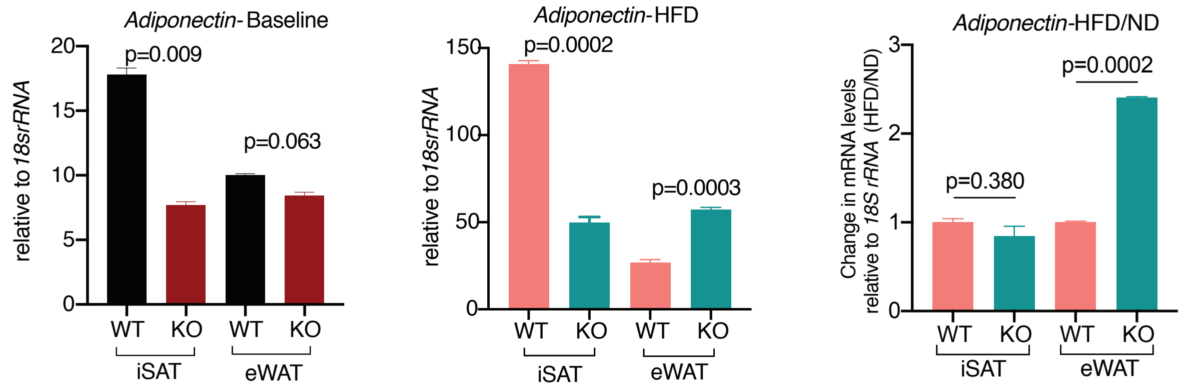

h

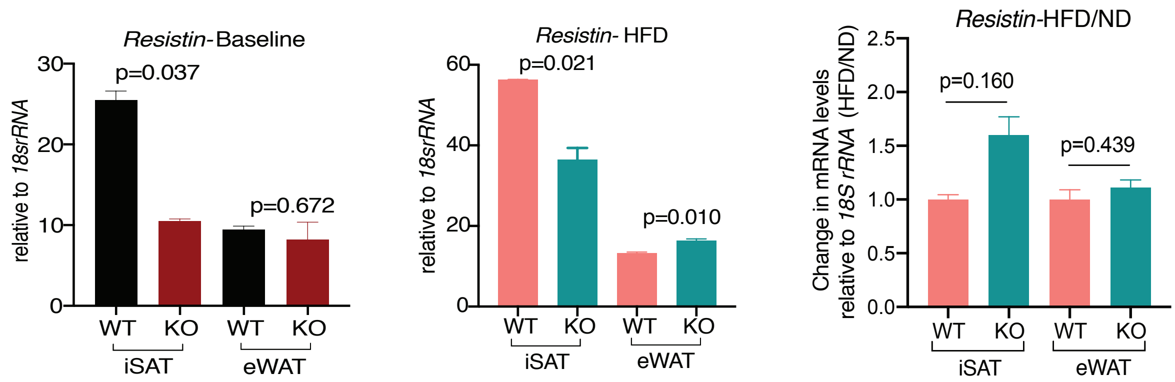

i

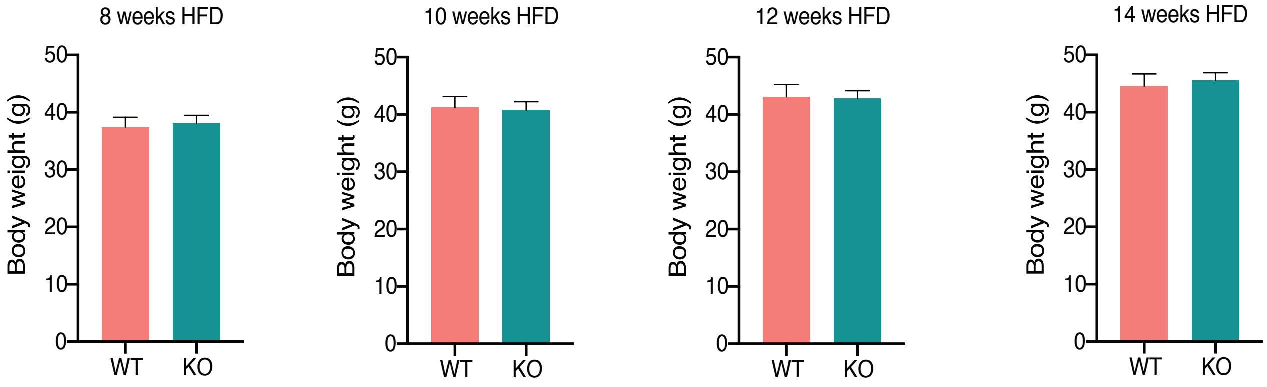

Supplementary Figure 5 Macroscopic appearance of harvested organs from ND- and HFD-fed mice, histological analysis of eWAT sections and adipokine expression changes

a) Macroscopic appearance of tissues harvested from mice on ND for 18 weeks - representative images.

b) µCT scans (transverse plane) of WT and KO mice fed HFD for 18 weeks- representative images

c) Gross appearance of tissues harvested from mice on HFD for 18 weeks - representative images.

d) Frequency distribution of adipocyte size in iSAT and eWAT sections of WT and KO male mice fed HFD for 18 weeks (n = 3)

e) Crown like structures quantification in eWATs from WT (n=9) and KO (n=6) mice fed HFD for 18 weeks

f) *Leptin* mRNA expression in adipose depots from male mice at baseline and upon long term HFD feeding and serum leptin levels at baseline (n=5 for WT and n=3 for KO)

g,h) *Adiponectin and resistin* mRNA expression in adipose depots from male mice at baseline and upon long term HFD feeding (n=3)

i) Whole body weight after 8, 10, 12, and 14 weeks of HFD feeding (n=11 for WT and n=8 for KO)

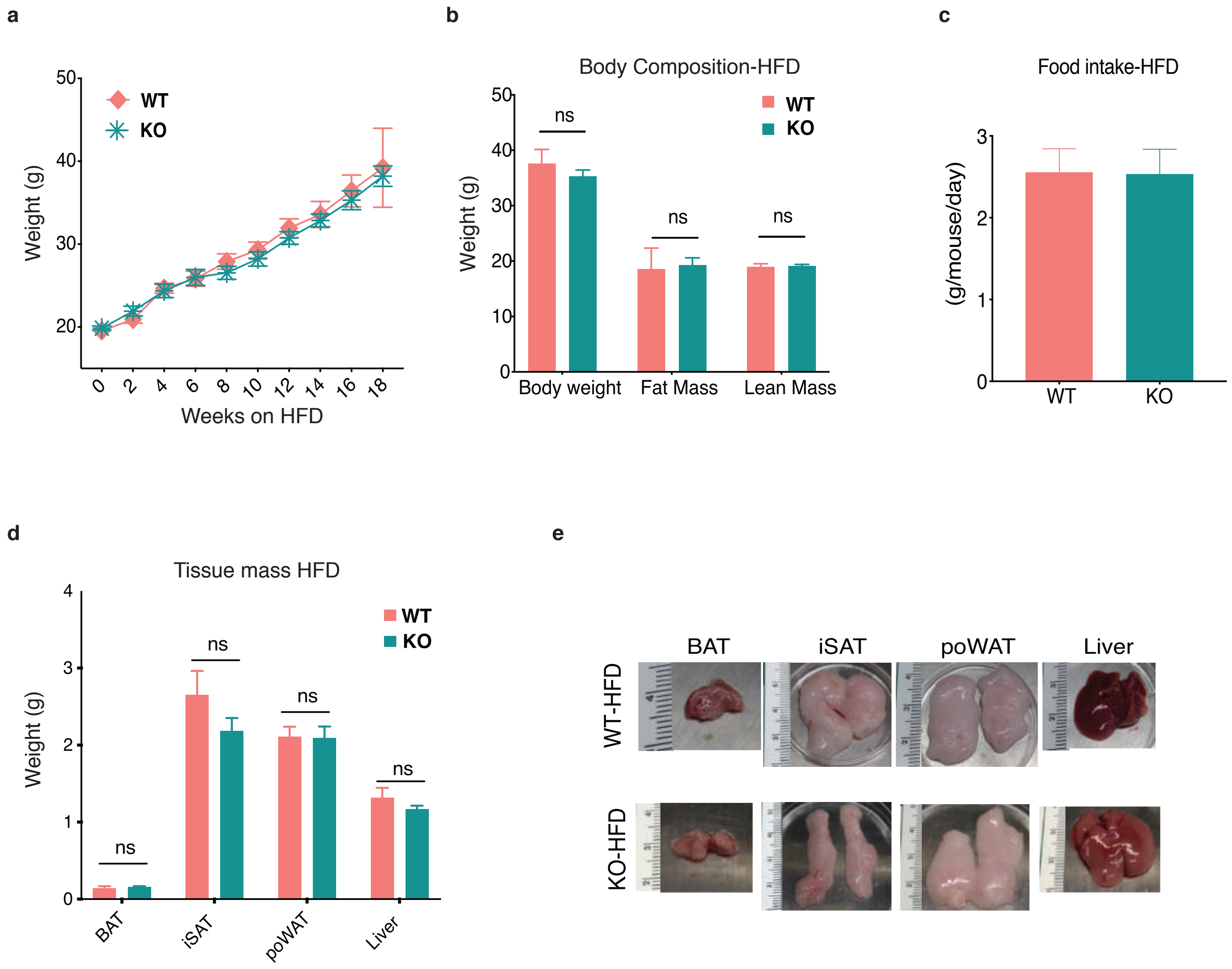

Supplementary Figure 6 Loss of AIF1L does not affect weight gain curves or adiposity in female mice upon long-term HFD feeding.
Female mice on HFD for 16-18 weeks

a) Total body weight curves of WT and KO mice fed high-fat diet starting at 8 weeks of age (n = 11 for WT, n = 13 for KO)
b) Body weight, fat mass and lean mass measured by MRI at the end of HFD feeding period; age - 26 weeks. (n = 7 for WT, n = 10 for KO)
c) Food intake over 4 weeks (n = 5)
d) Brown (BAT), inguinal subcutaneous (iSAT), peri-ovarian (poWAT) white adipose depot, and liver mass. (n = 7 for WT, n = 10 for KO)
e) Gross appearance of tissues- representative images

Data are represented as mean ± SEM. ns - not significant.

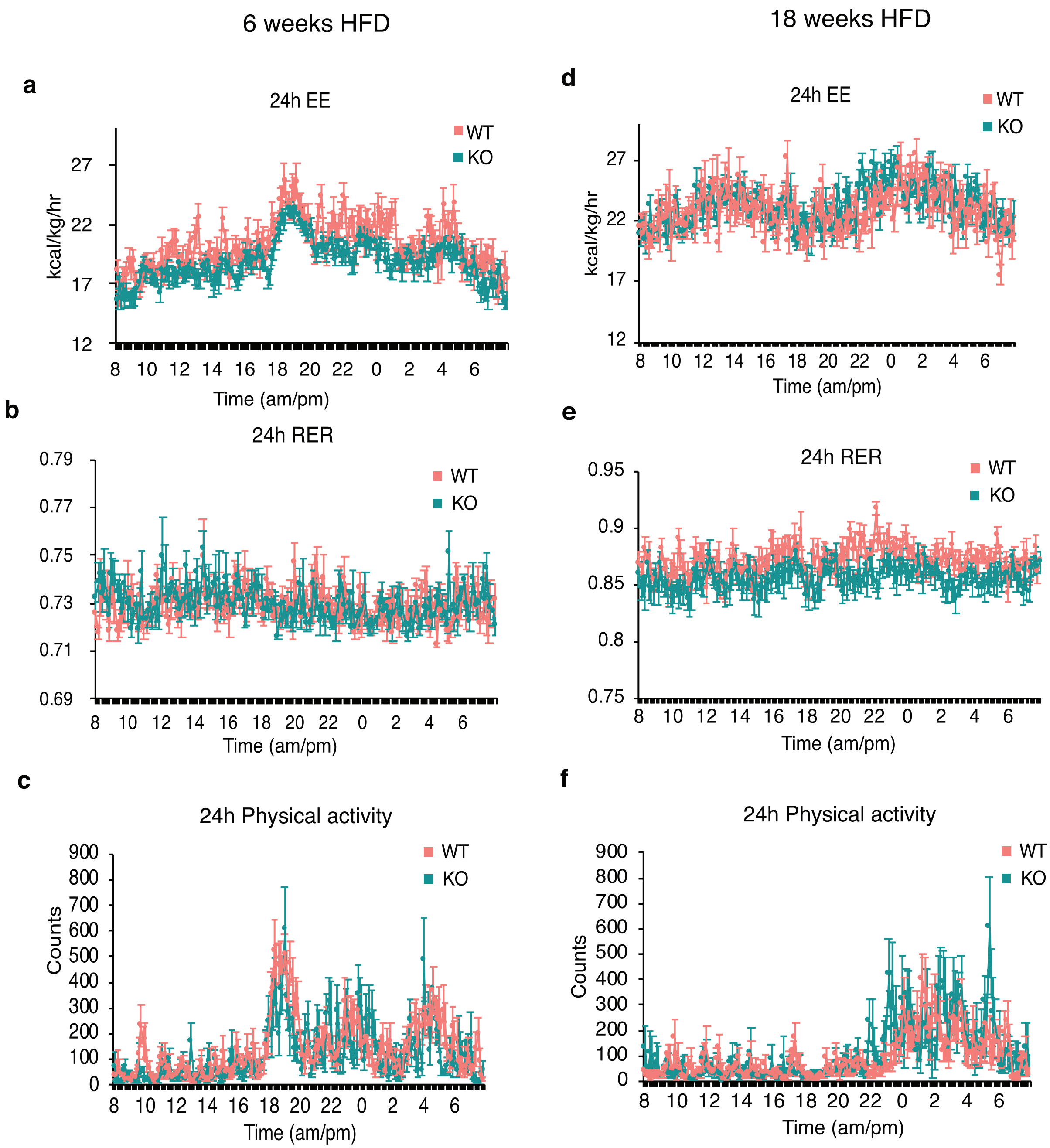

Supplementary Figure 7 Metabolic profile of WT and AIF1L deficient mice over a 24h period, upon short- and long-term HFD feeding

a-c) Energy expenditure, RER, and Physical activity measurements over a 24 h period in metabolic cages for WT and KO mice fed HFD for 6 weeks (n = 3 for each genotype)

d-f) Energy expenditure, RER, and Physical activity measurements over a 24 h period in metabolic cages for WT and KO mice fed HFD for 18 weeks (n = 3 for each genotype)

Data are represented as mean ± SEM. ns- not significant.

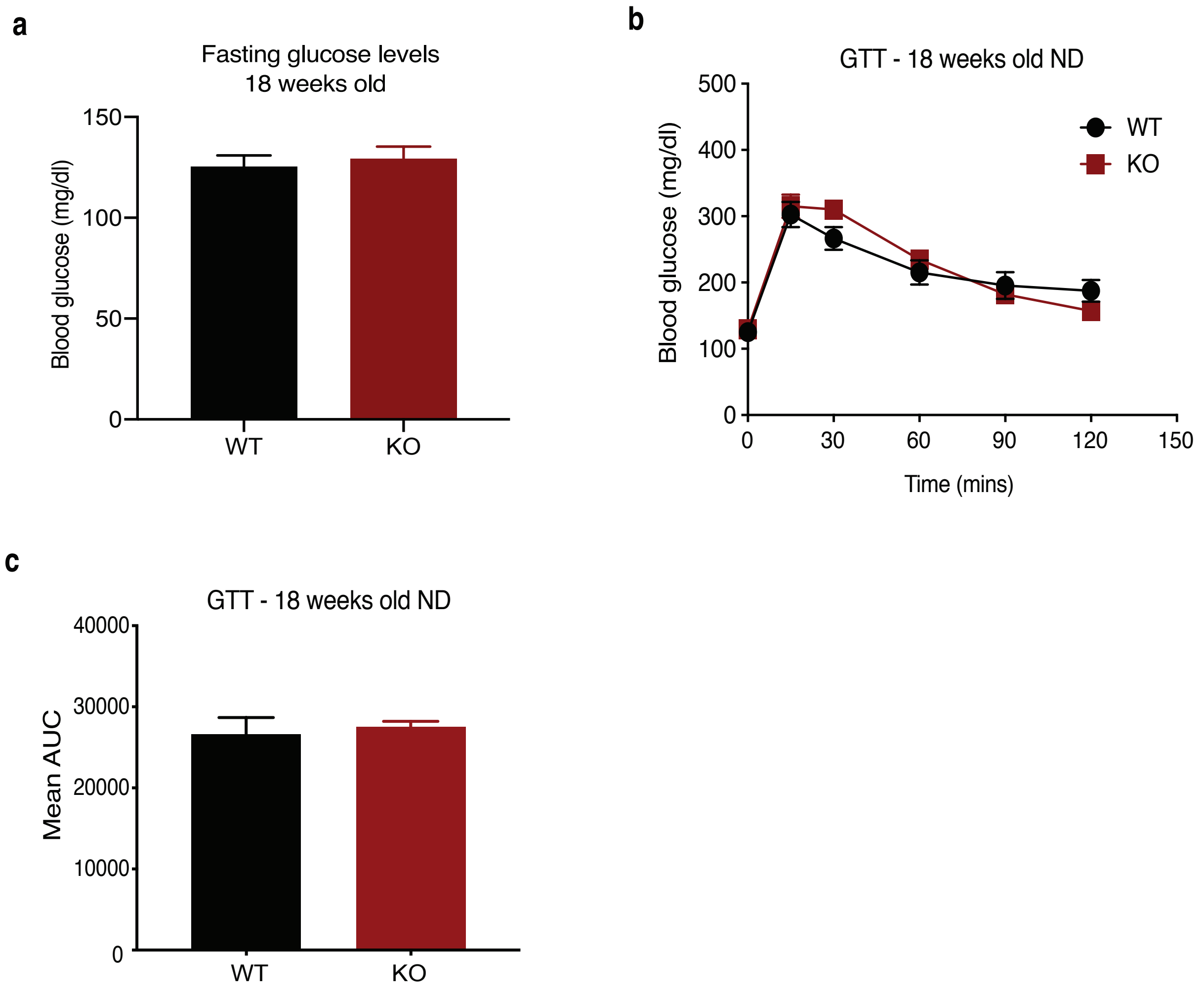

Supplementary Figure 8. 18 weeks old WT and AIF1L deficient mice have similar response to glucose challenge

(a–c) 18 weeks old male mice on ND

a) Blood glucose levels in WT (n = 3) and KO (n = 4) mice after overnight fasting.
b) IPGTT (intra peritoneal glucose tolerance test) with 1g/kg (of body weight) of glucose.
c) Quantification of GTT as mean area under curve (AUC).

Data are represented as mean ± SEM. ns - not significant.

Uncropped blots for Figure 1a- BAT

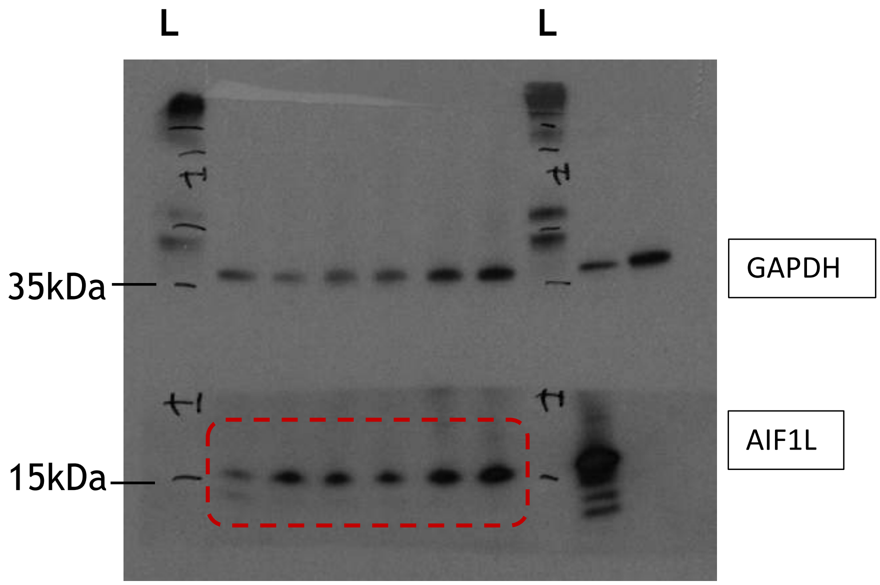

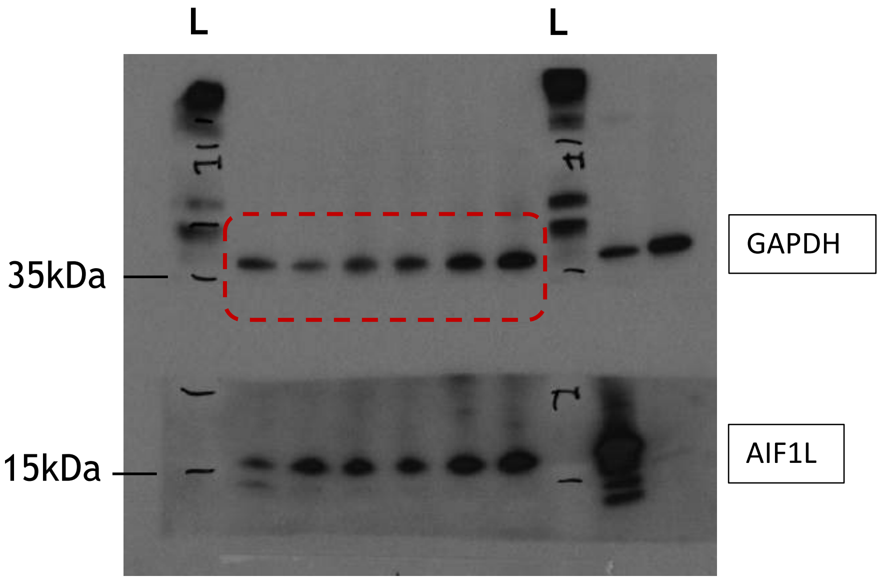

Cropped along the dotted lines

L Ladder

Uncropped blots for Figure 1a- SAT

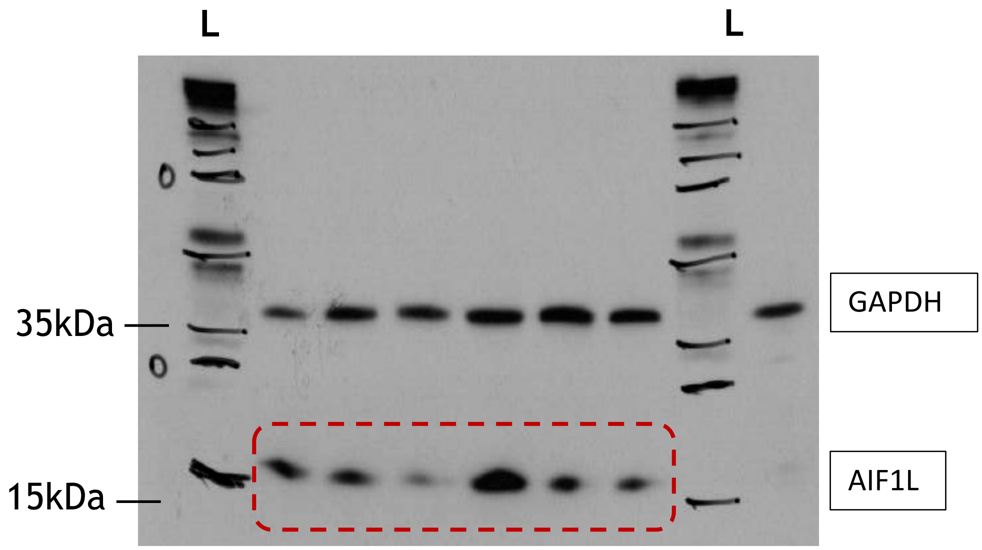

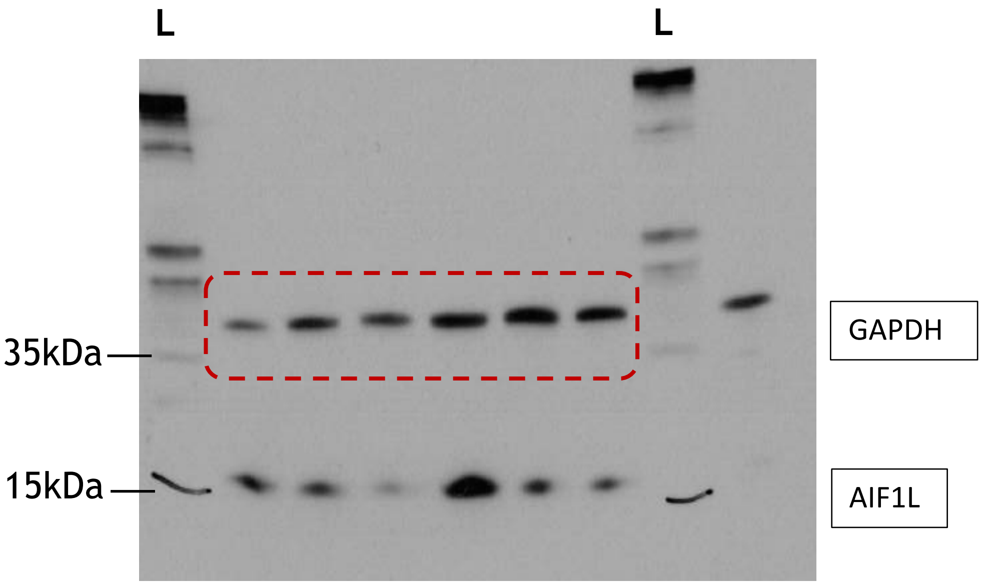

Cropped along the dotted lines

L Ladder

Uncropped blots for Figure 1a- eWAT Males

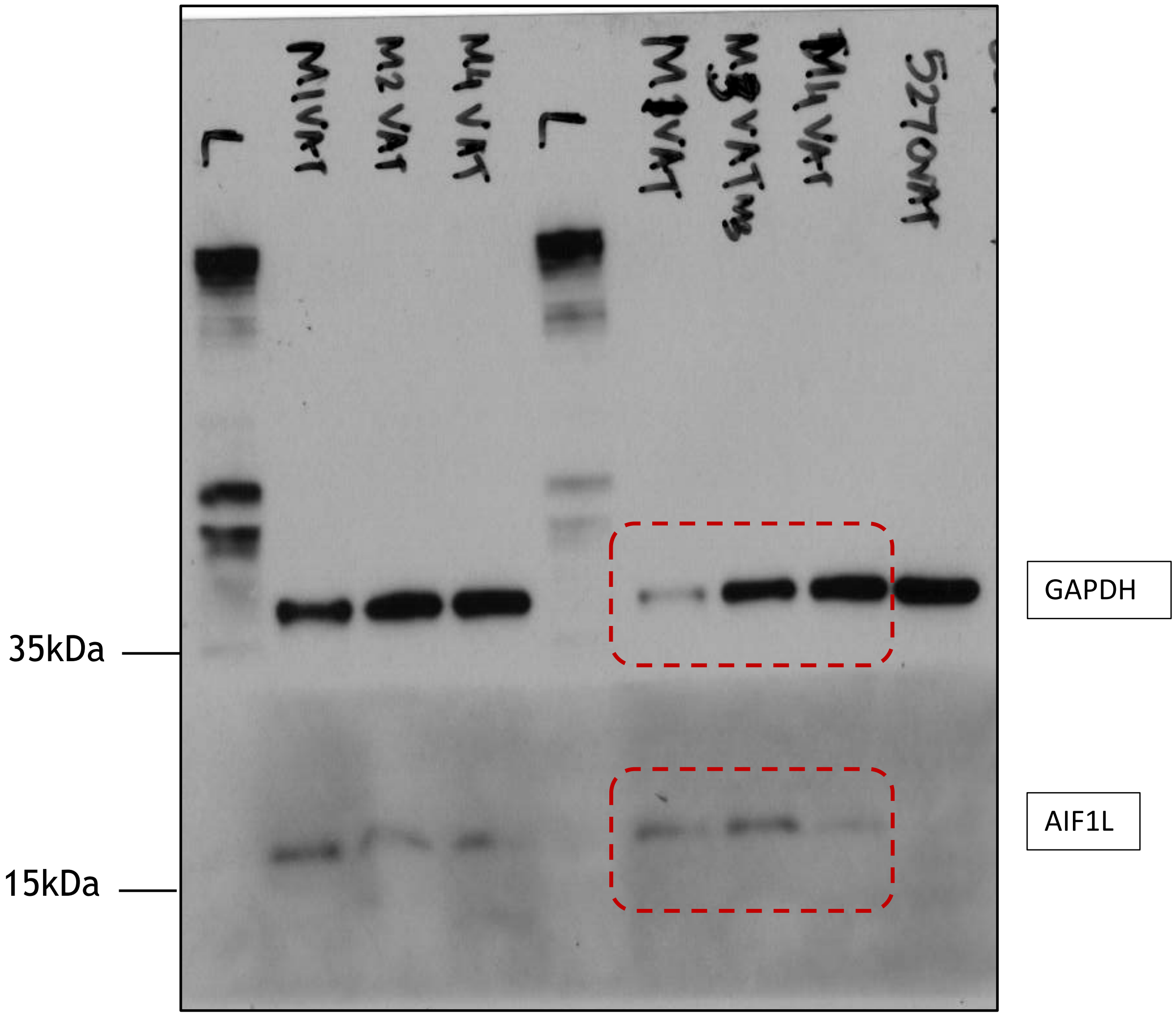

Cropped along the dotted lines

L Ladder

Uncropped blots for Figure 1a- eWAT-Females

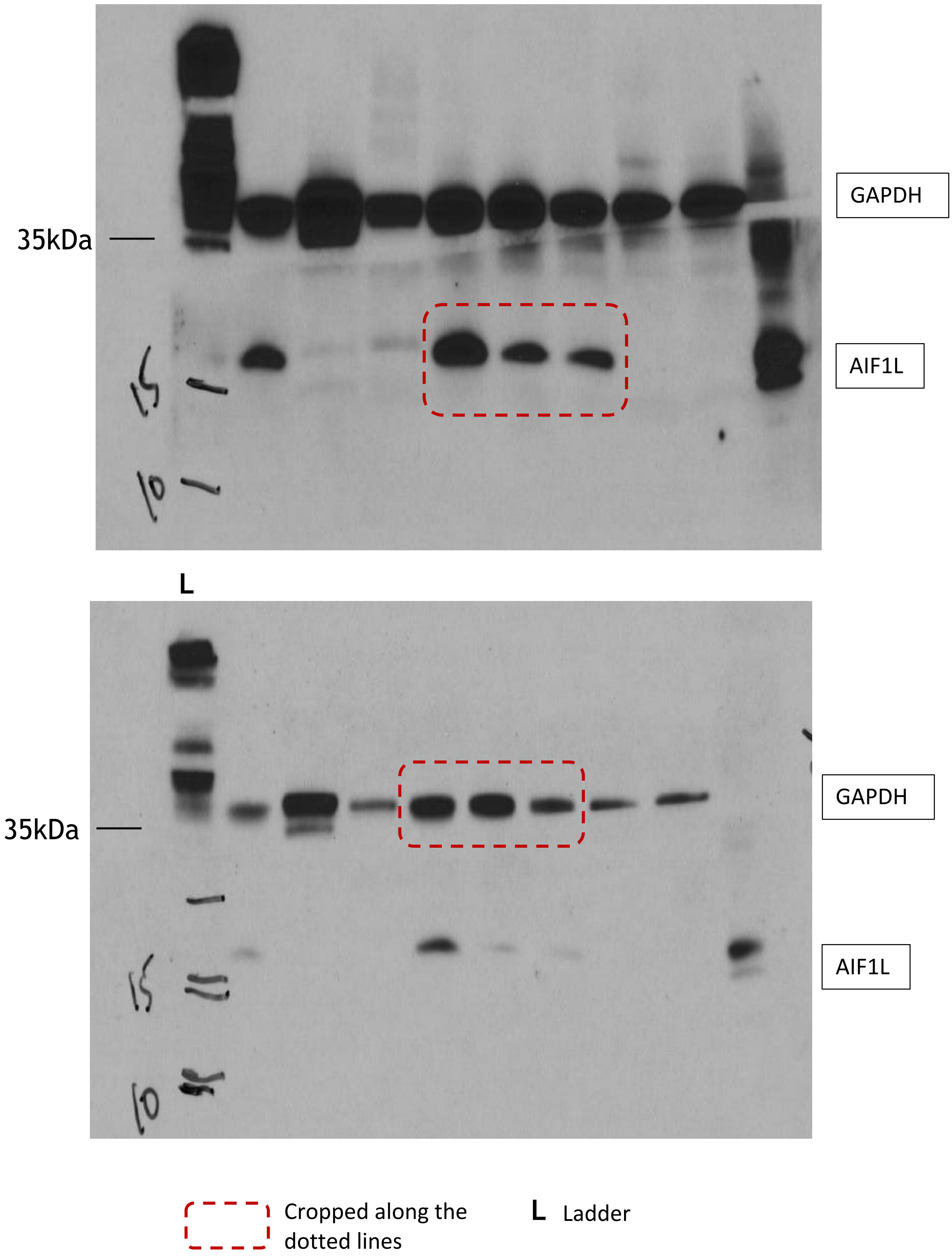

Uncropped blot #2 used for VAT quantification and comparison between males and females -

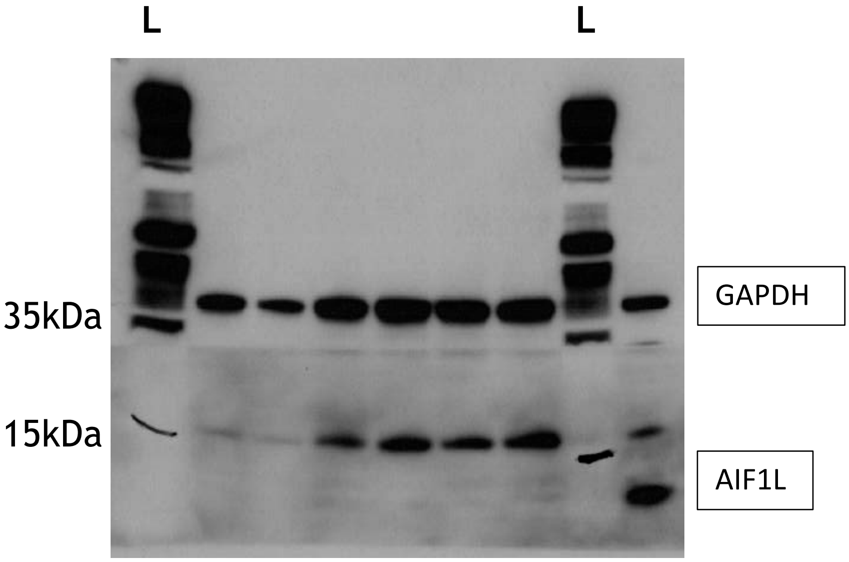

Uncropped blots for Figure 1b- Kidney

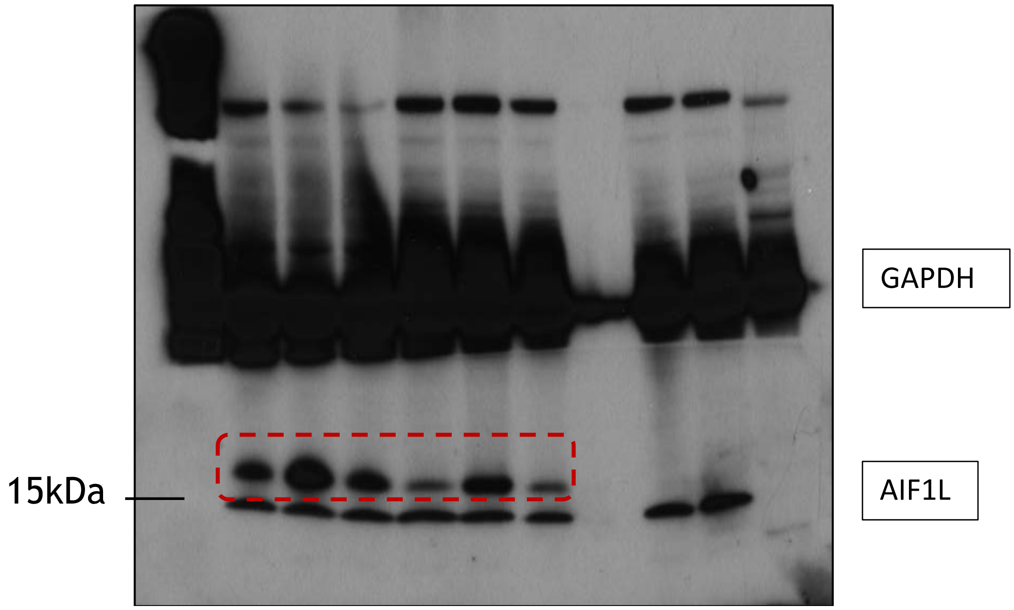

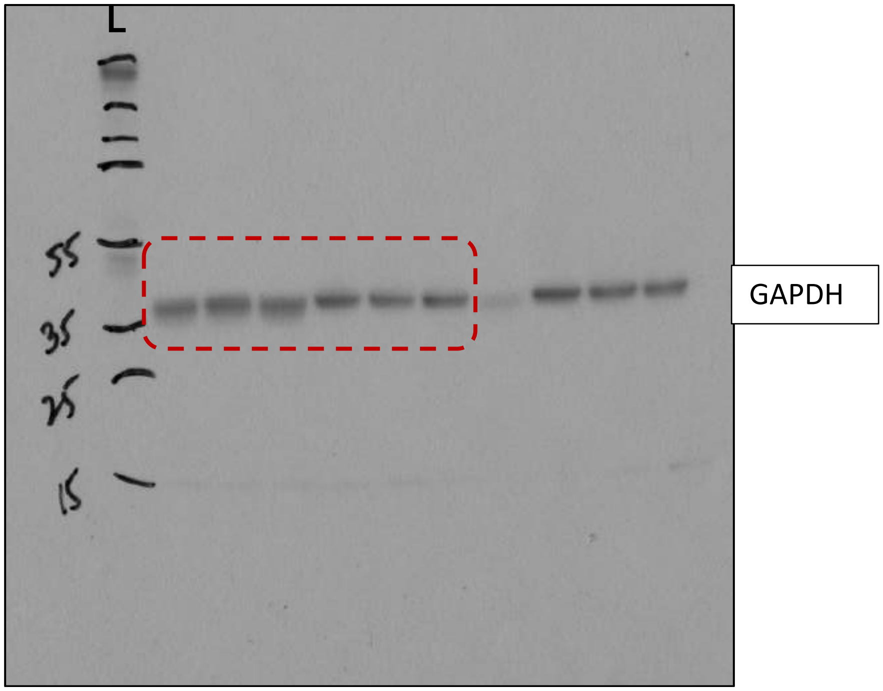

Cropped along the dotted lines

L Ladder

Uncropped blots for Figure 1b- Brain

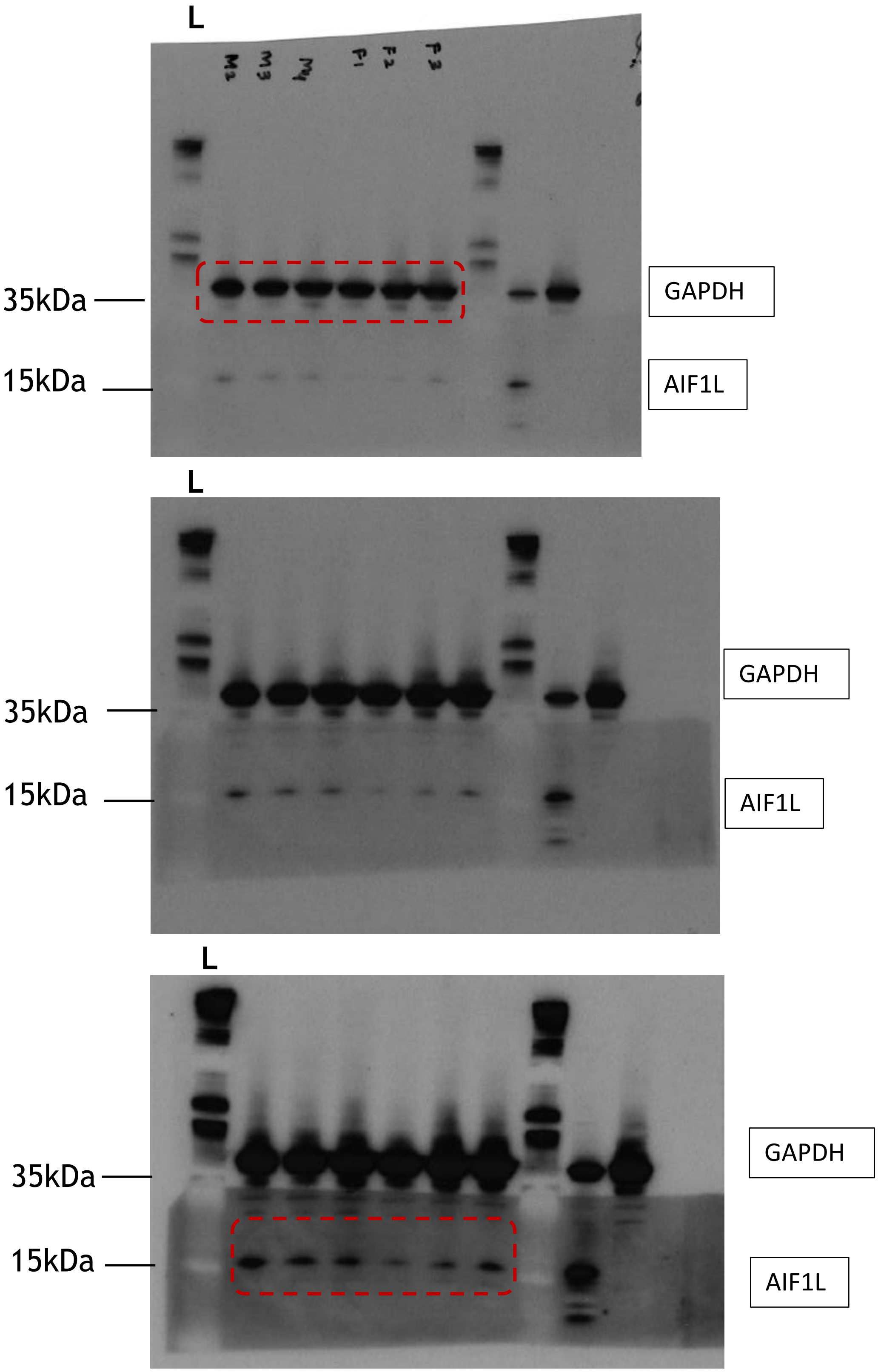

Cropped along the dotted lines

L Ladder

Uncropped blots for Figure 1b- Lung

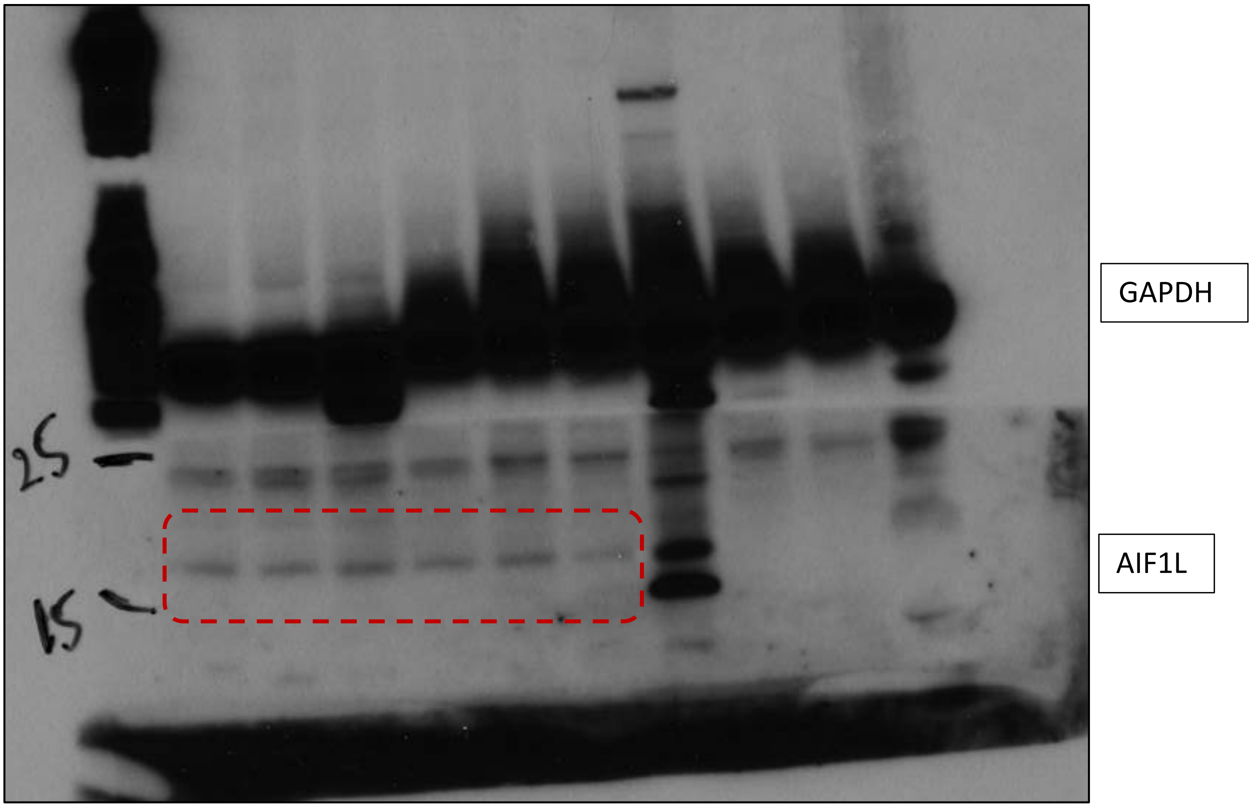

GAPDH

AIF1L

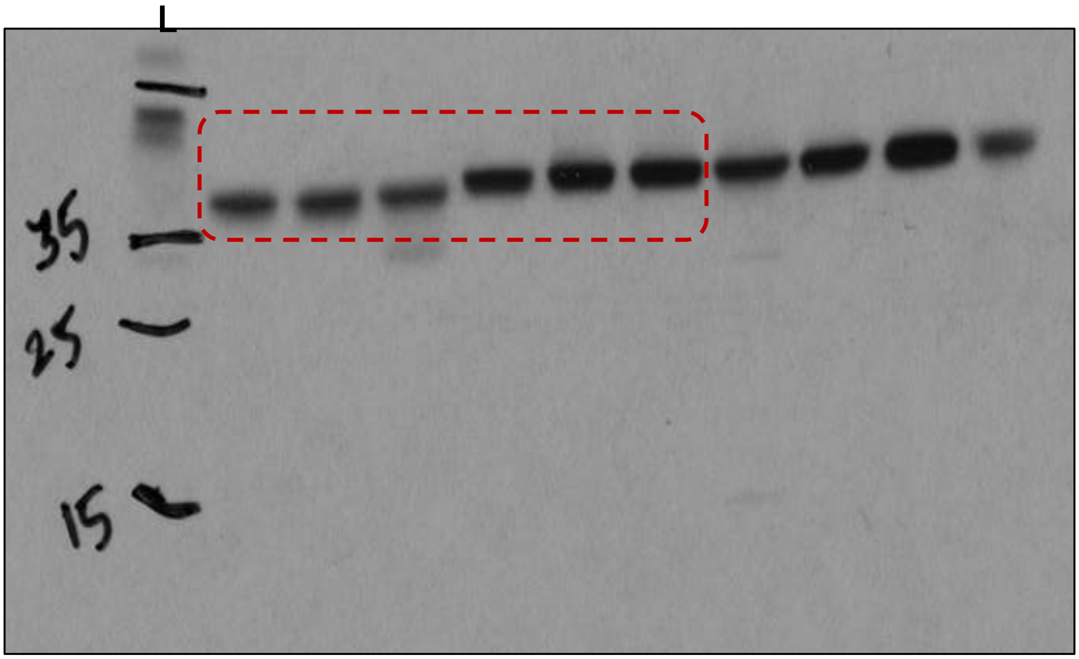

GAPDH

Cropped along the dotted lines

L Ladder

Uncropped representative blots used in Figure 1c- BAT

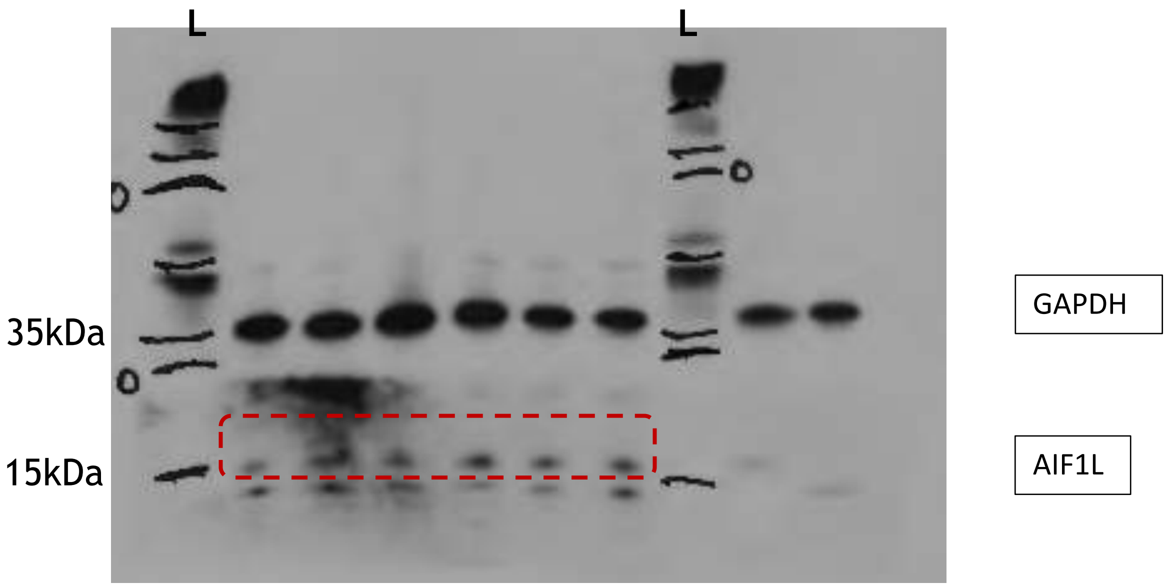

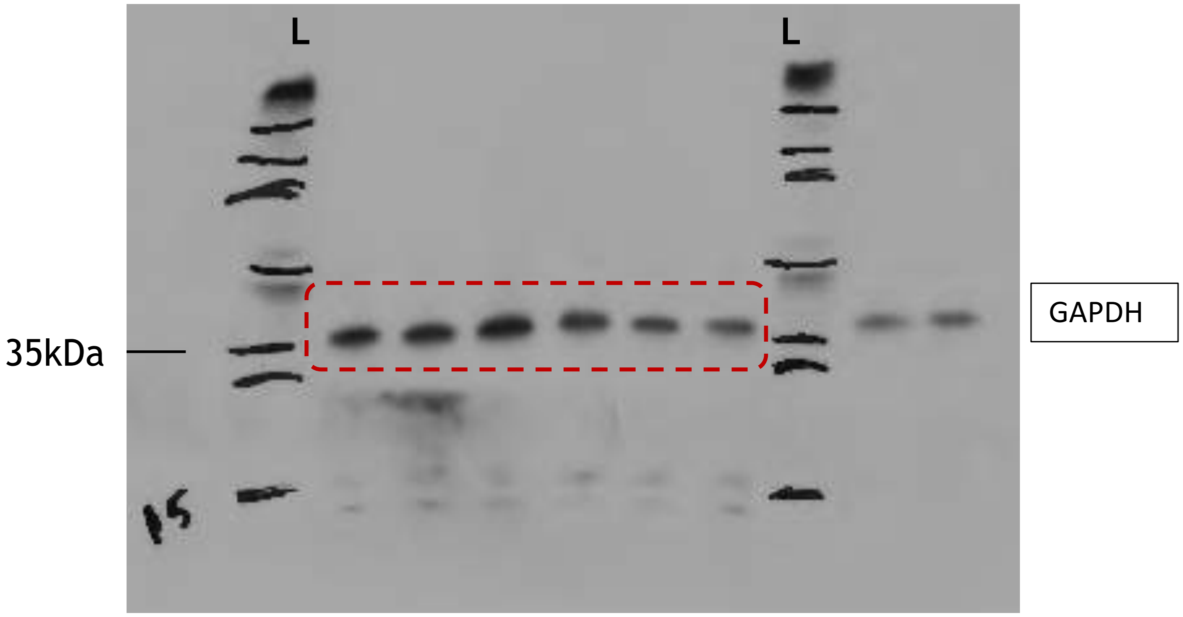

Cropped along the dotted lines

L Ladder

Uncropped blots for Figure 1c- iSAT

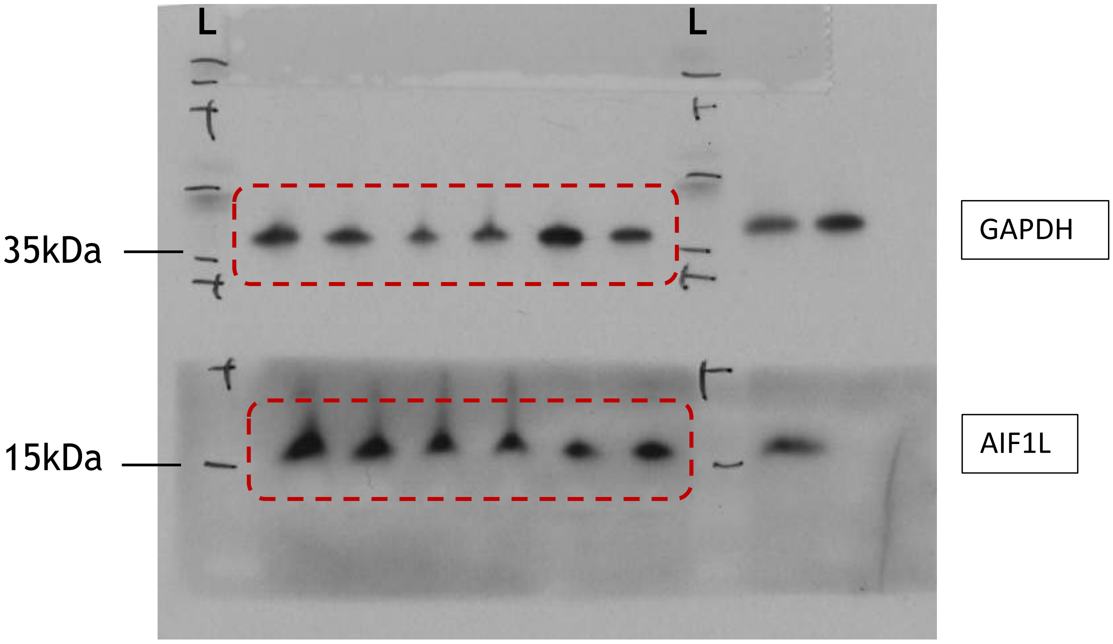

Uncropped blots for Figure 1c- eWAT

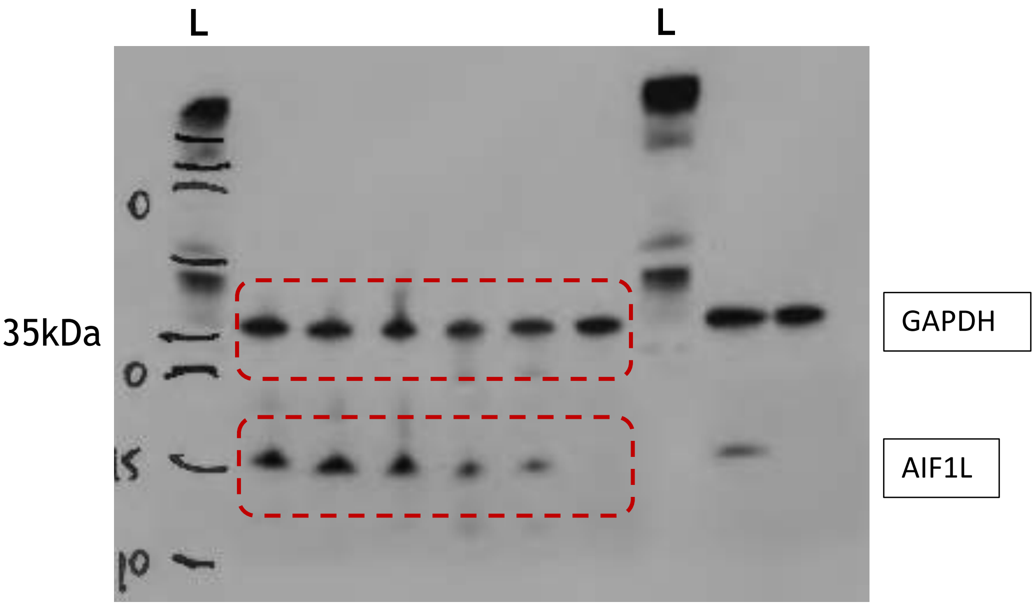

Cropped along the dotted lines

L Ladder

Uncropped blots for Figure 2a- BAT

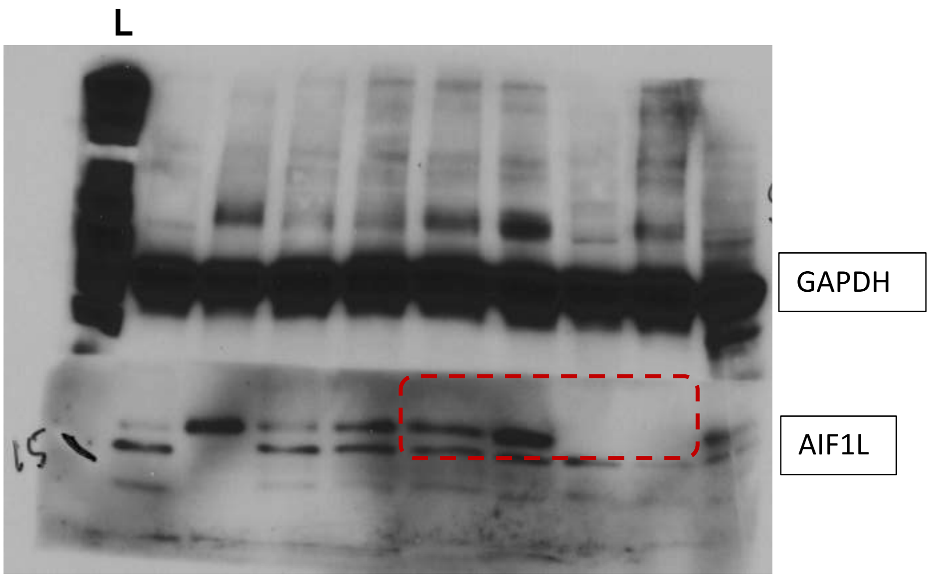

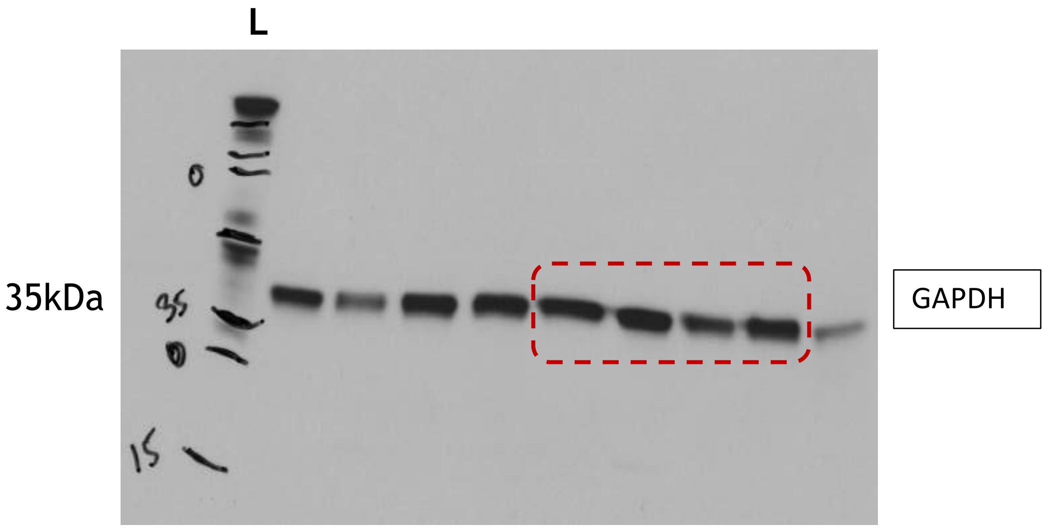

Cropped along the dotted lines

L Ladder

Uncropped blots for Figure 2a- iSAT

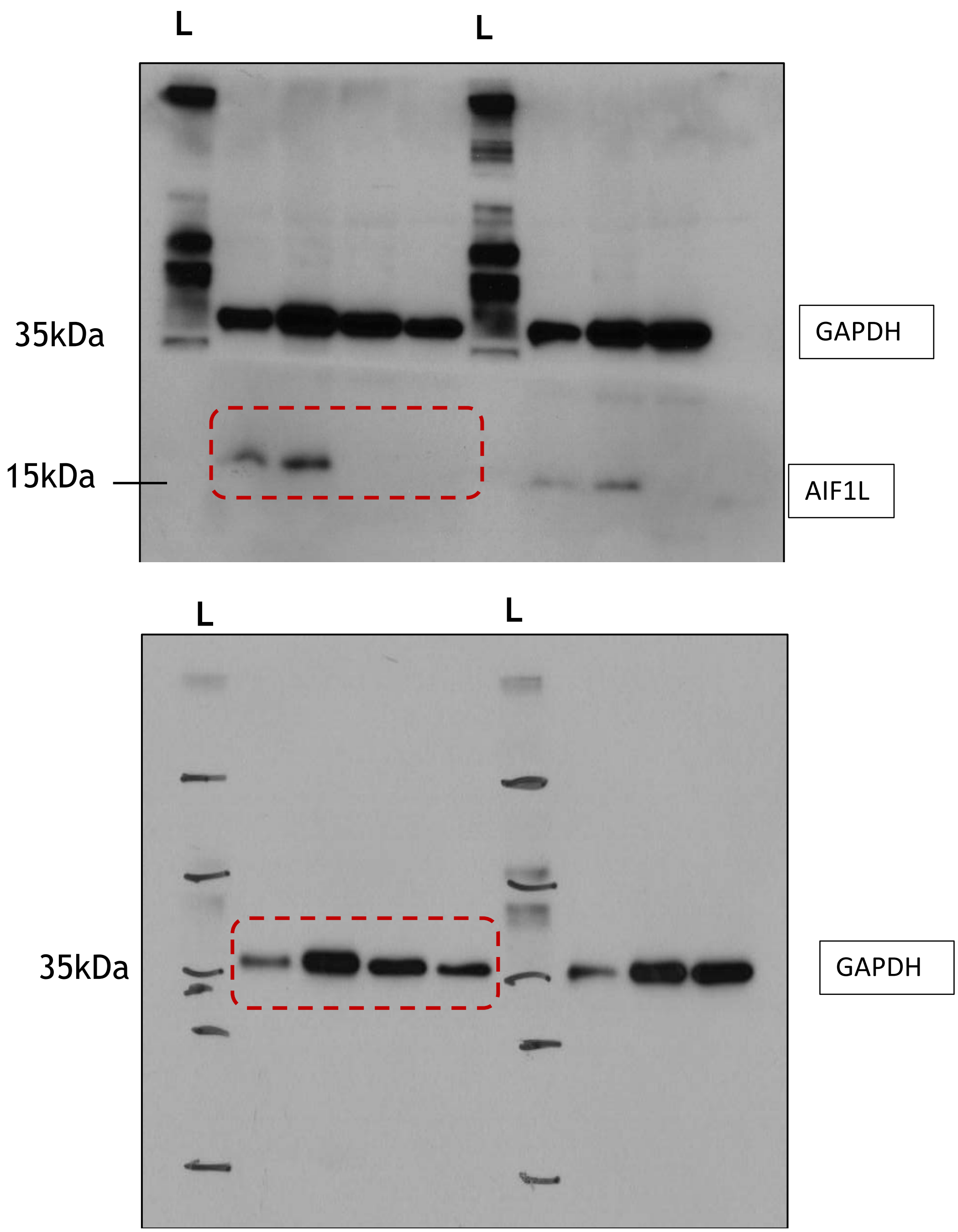

Cropped along the dotted lines

L Ladder

Uncropped blots for Figure 2a- eWAT

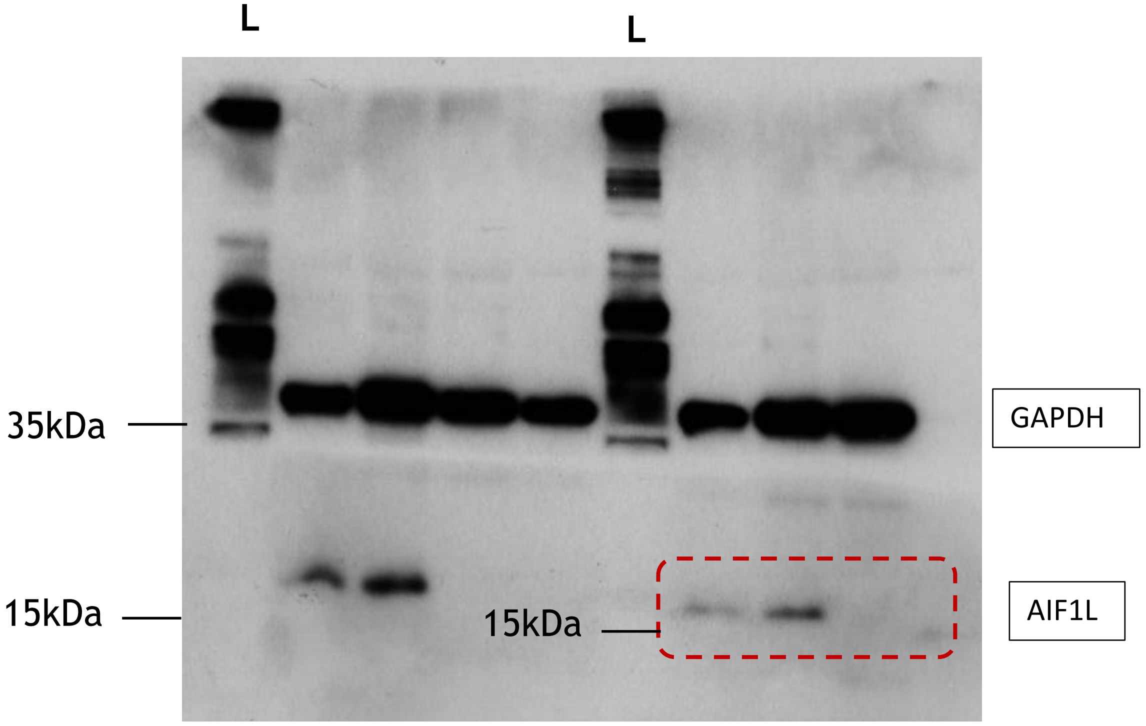

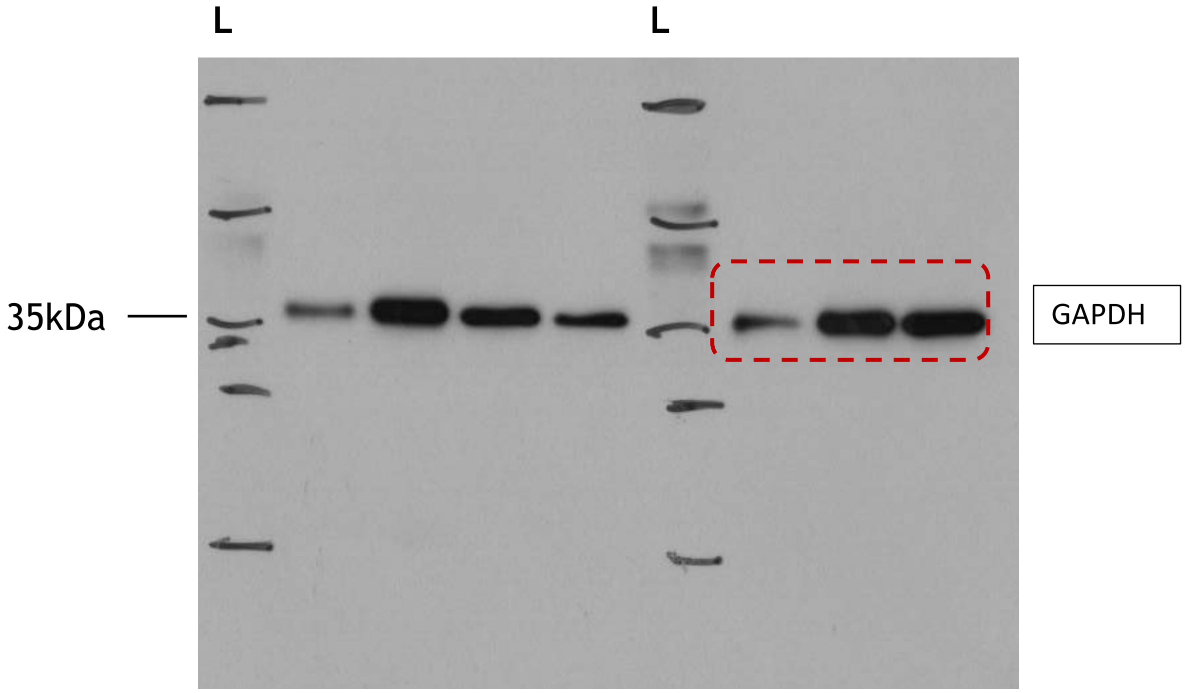

Cropped along the dotted lines

L Ladder

Uncropped blots for Figure 2a- Brain

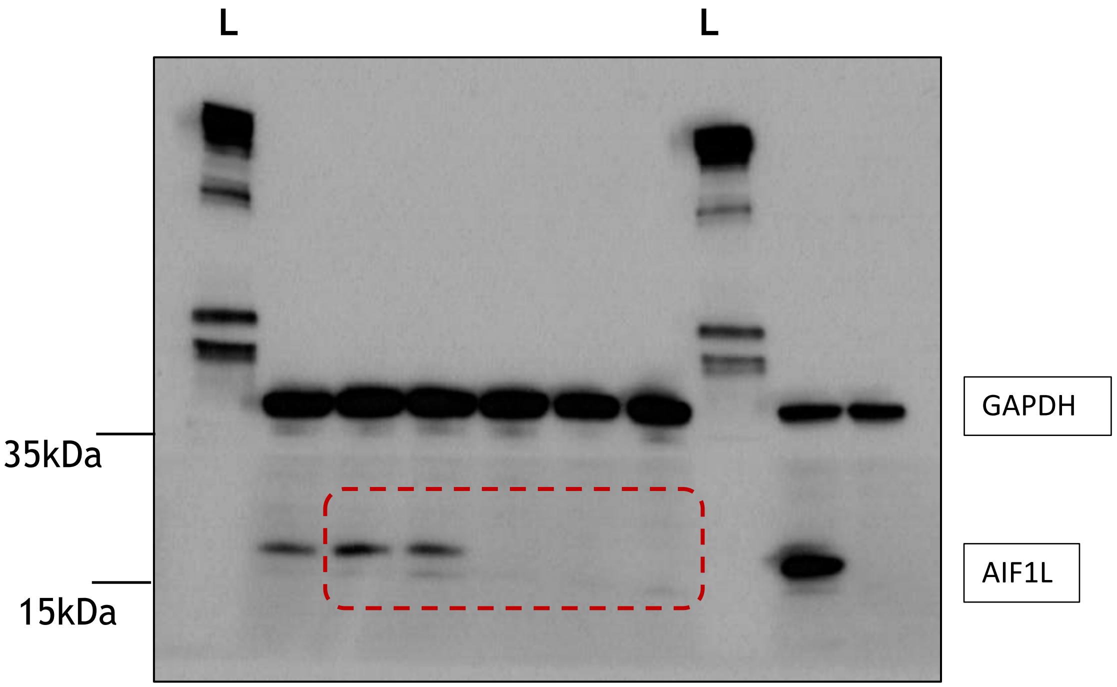

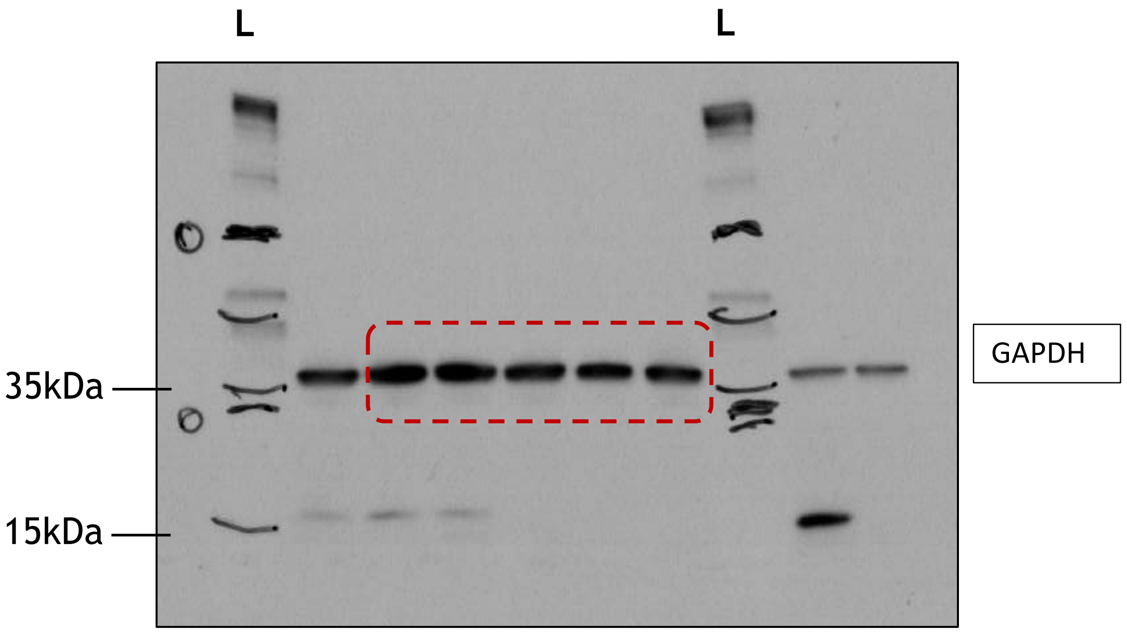

Cropped along the dotted lines

L Ladder

Uncropped blots for Supplementary Figure 1a

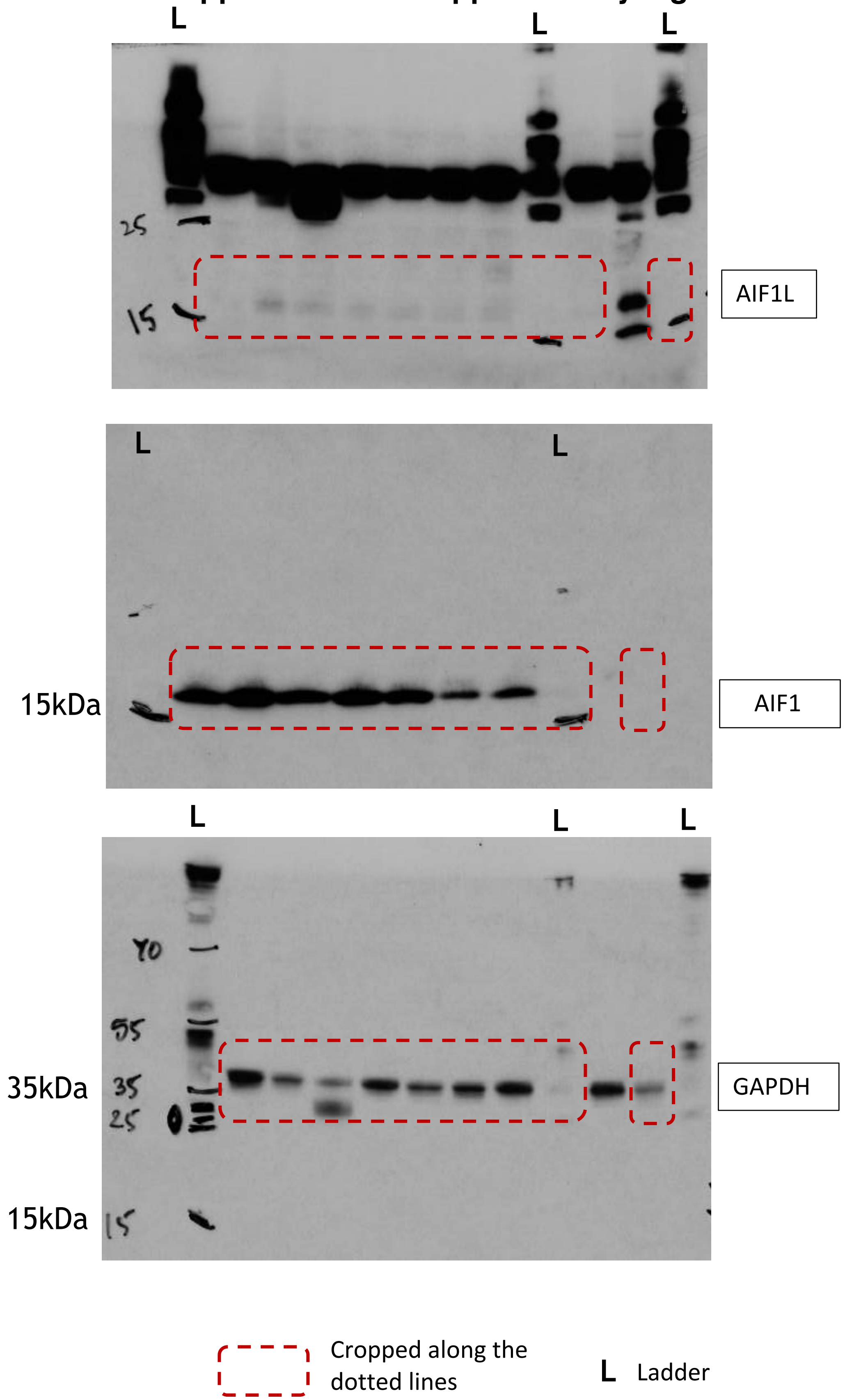

Uncropped blots for Supplementary Figure 1b- 3T3L1 cells

L L

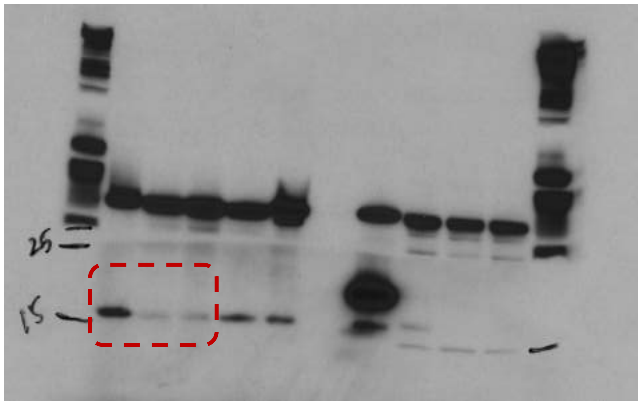

AIF1L

Shorter exposure

L L

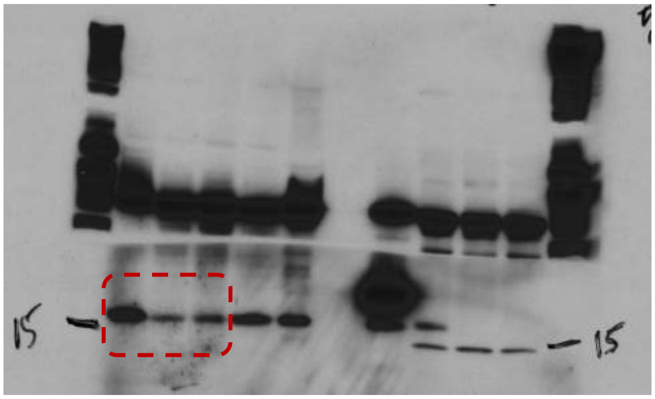

AIF1L

Longer exposure

L L

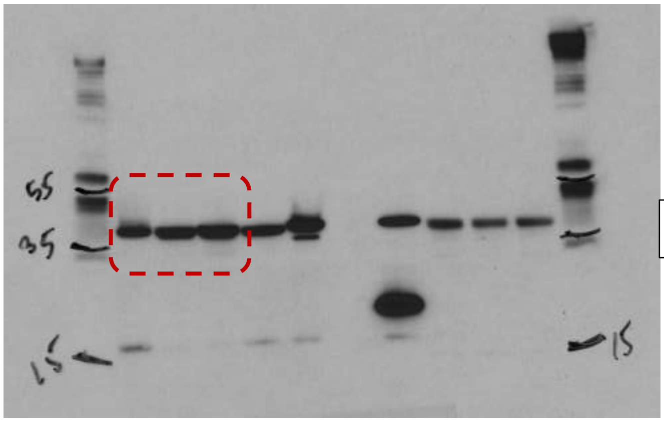

GAPDH

Cropped along the dotted lines

L Ladder

Uncropped blots for Supplementary Figure 1c- RAW 264.7 cells

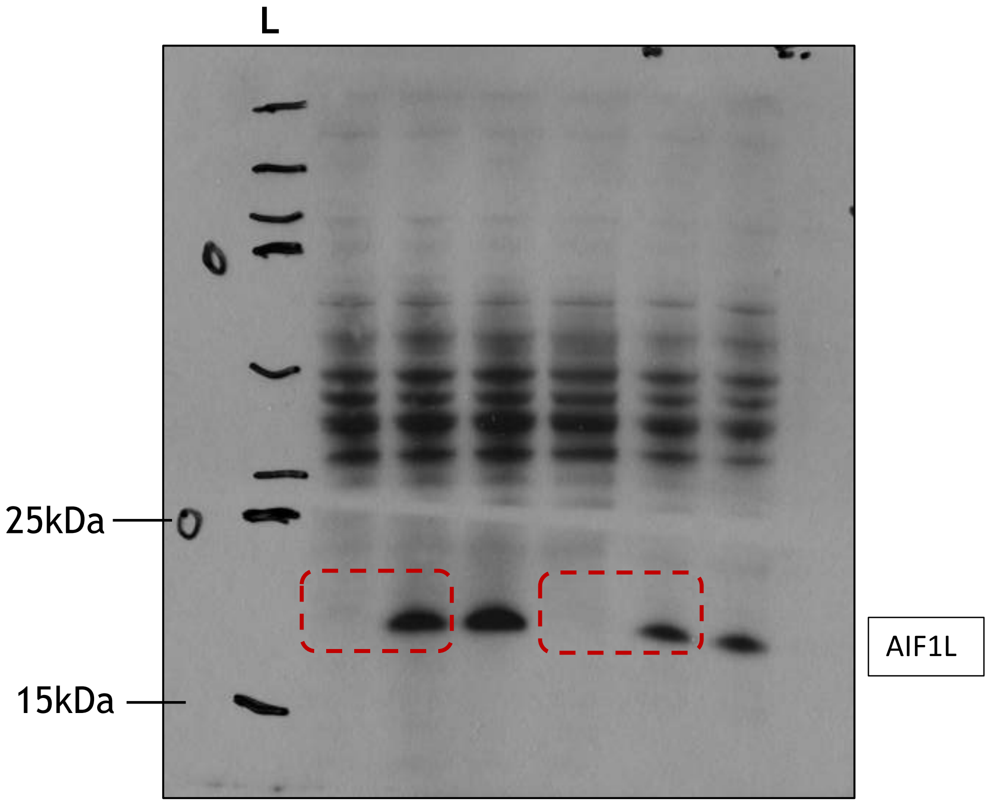

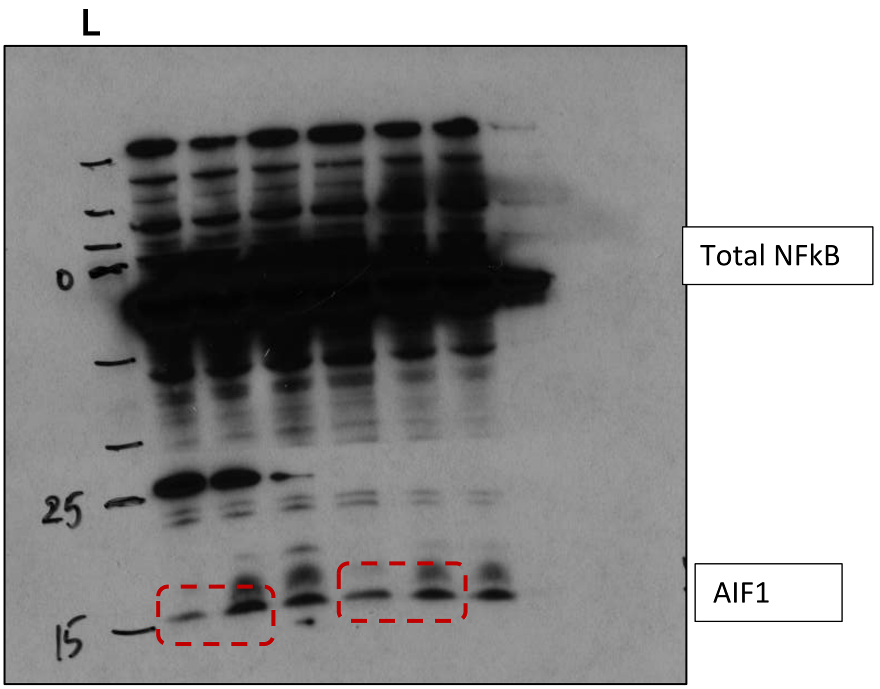

Cropped along the dotted lines

L Ladder

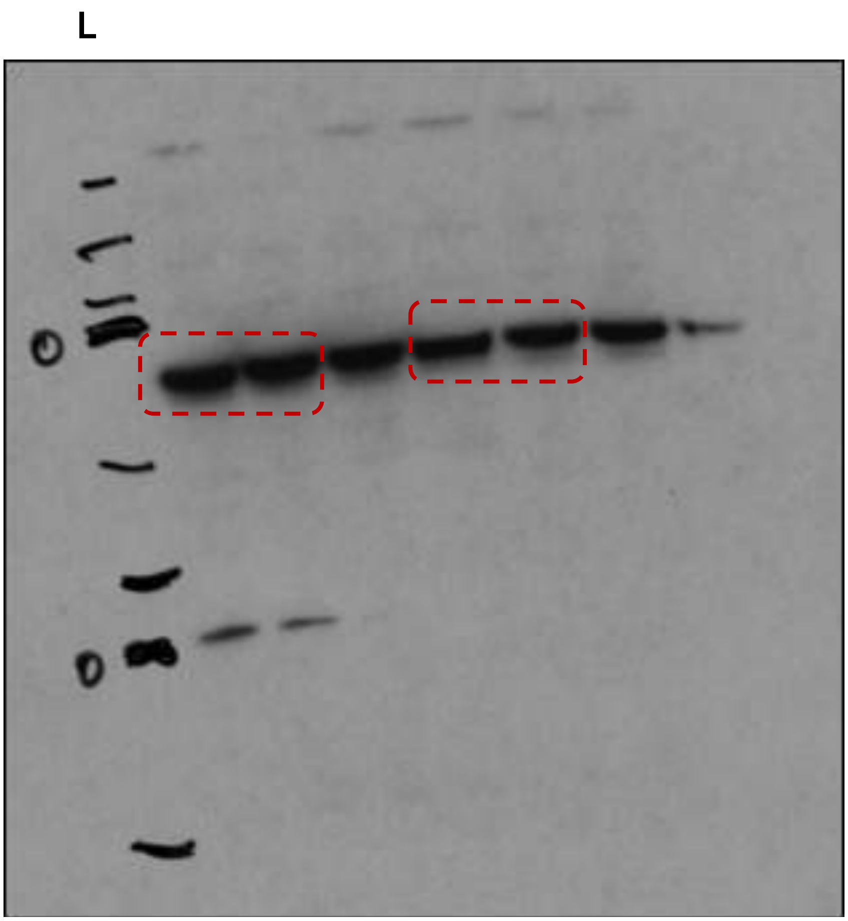

Total NFkB

Cropped along the dotted lines

L Ladder

Uncropped blots for Supplementary Figure 2a- Liver

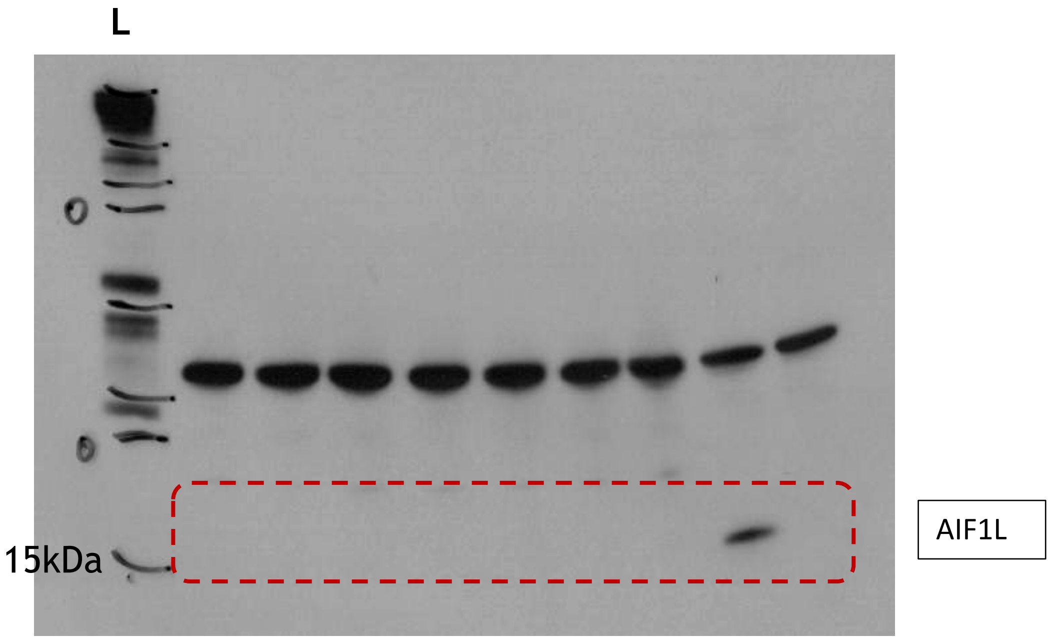

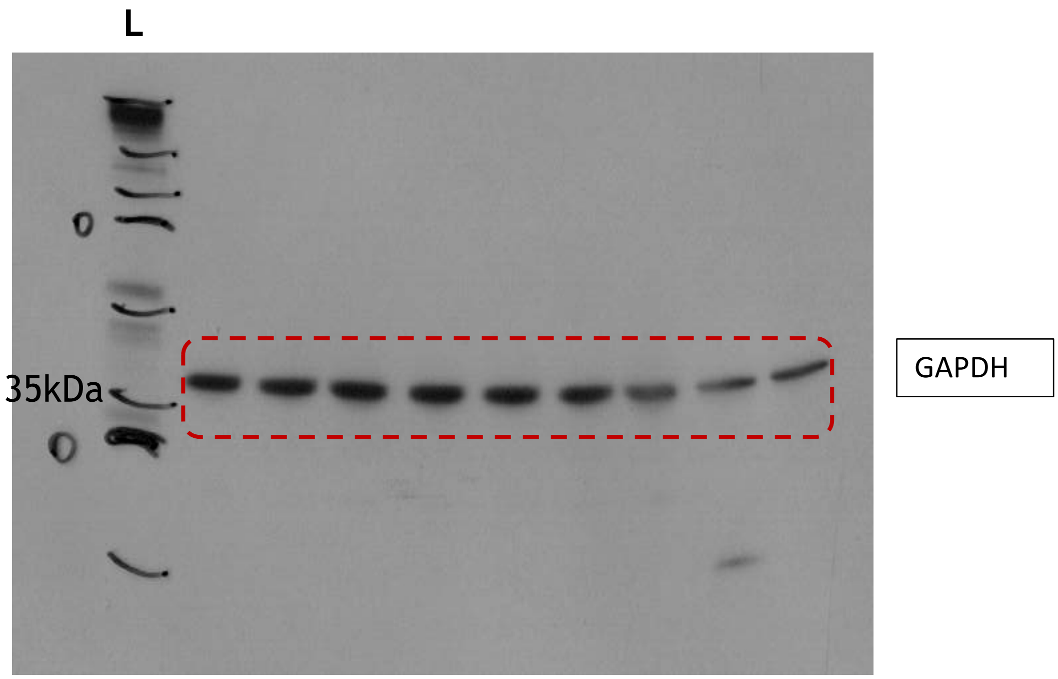

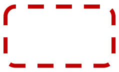

Cropped along the dotted lines

L Ladder

Uncropped blots for Supplementary Figure 2a- Heart

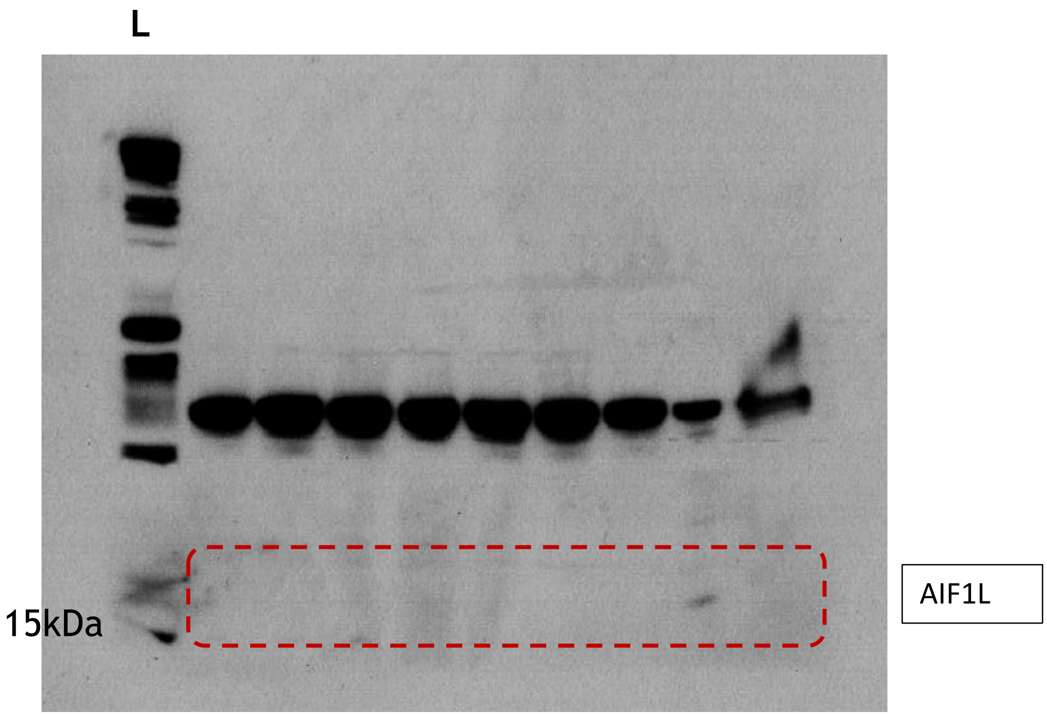

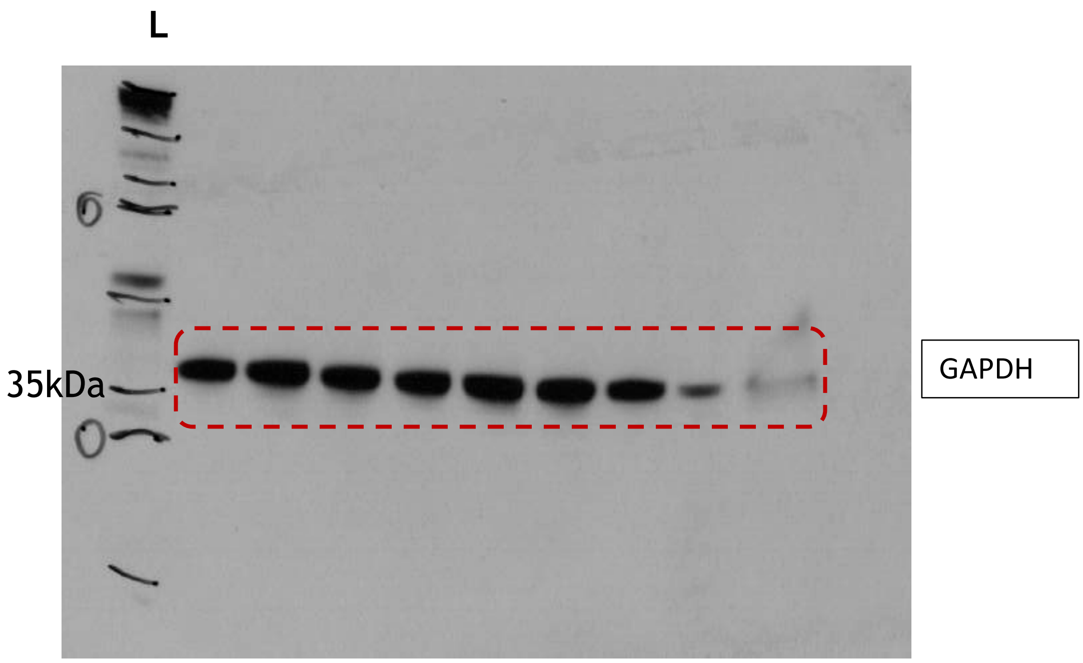

Cropped along the dotted lines

L Ladder

Uncropped blots for Supplementary Figure 2a- Skeletal muscle

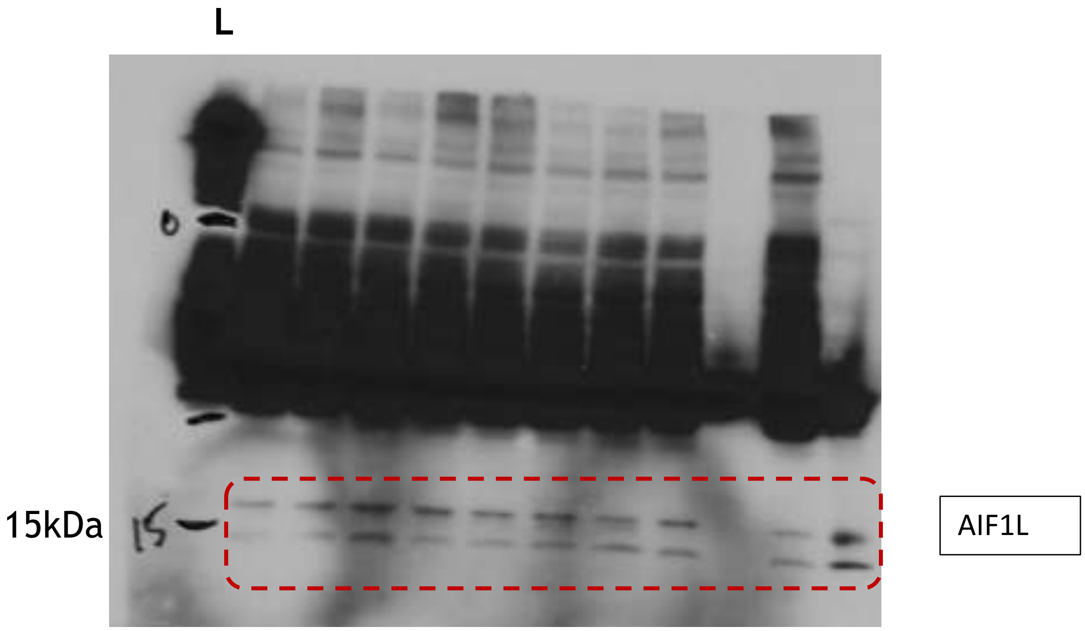

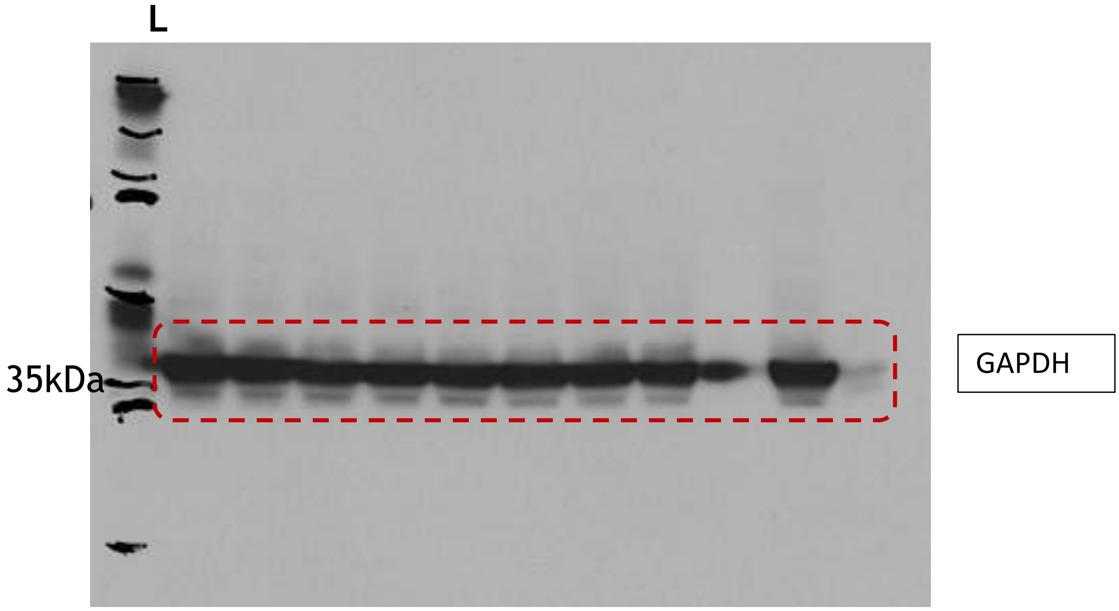

Cropped along the dotted lines

L Ladder

Uncropped blots for Supplementary Figure 4a- BAT

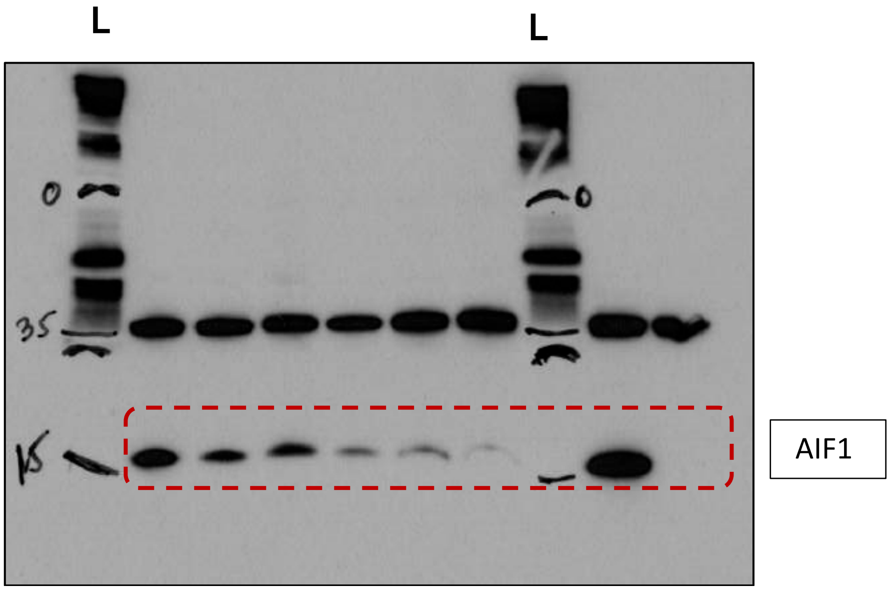

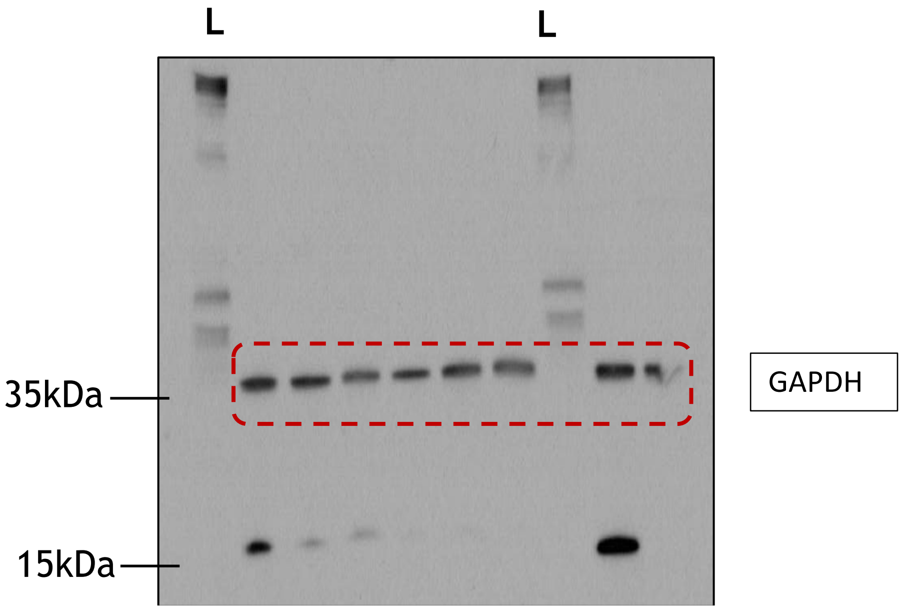

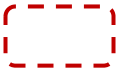

L Ladder

Uncropped blots for Supplementary Figure 4a- VAT

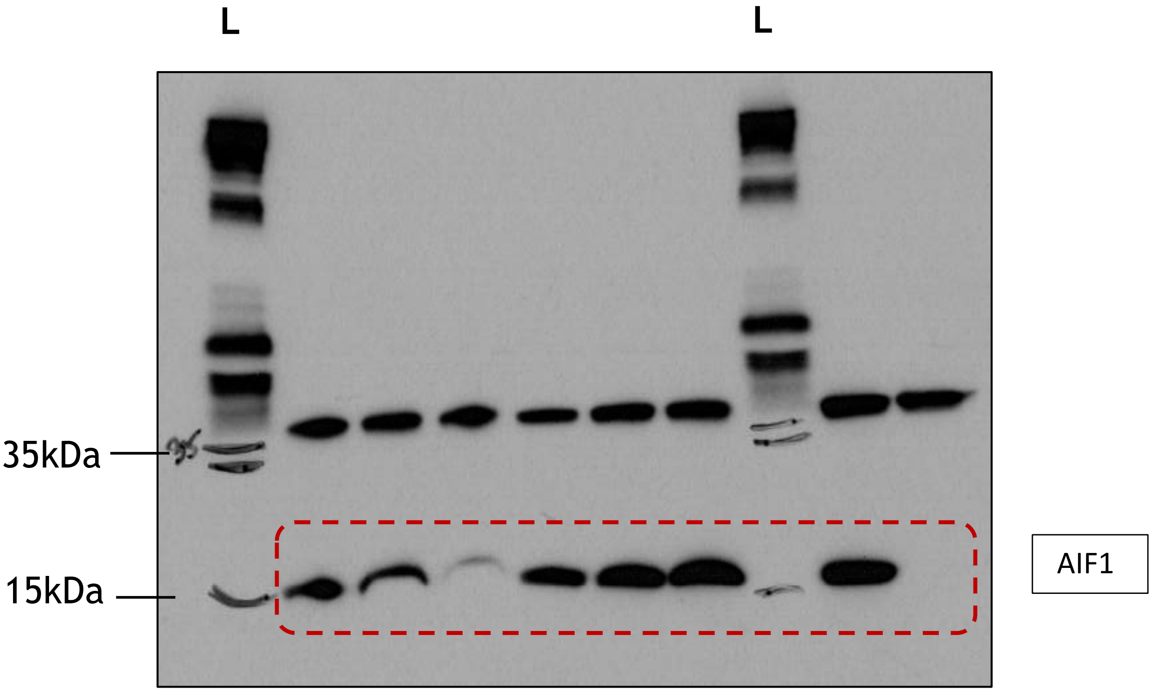

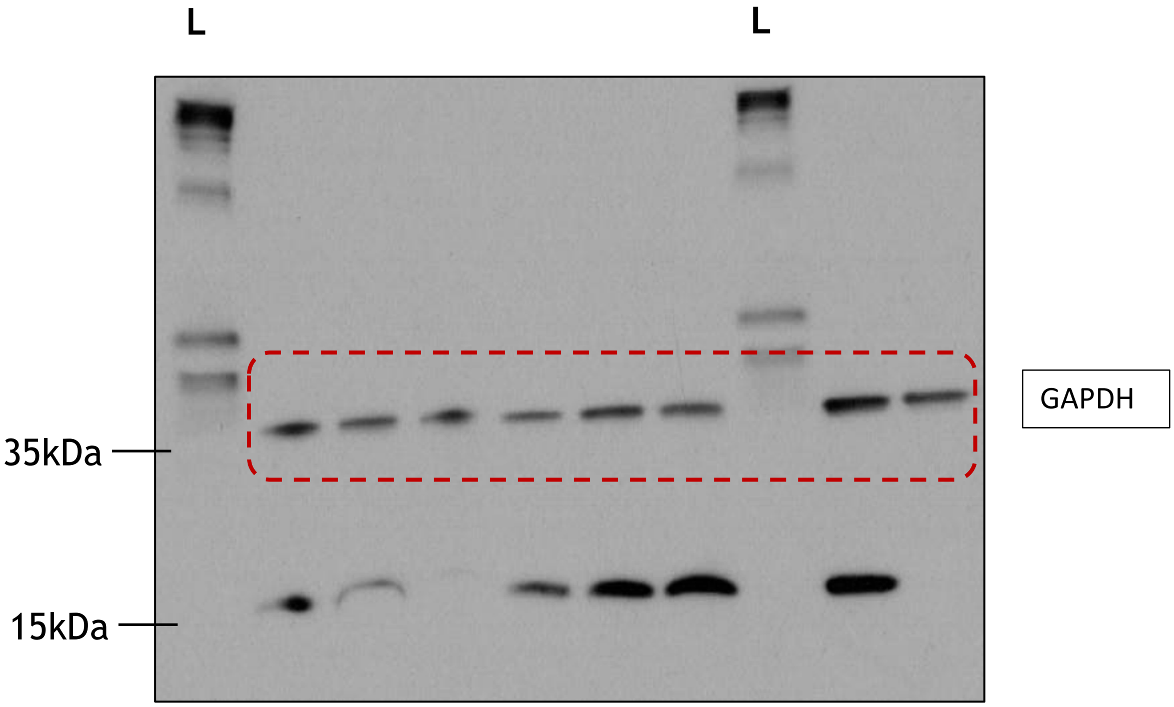

Cropped along the dotted lines

L Ladder

Uncropped blots for Supplementary Figure 4a- Brain

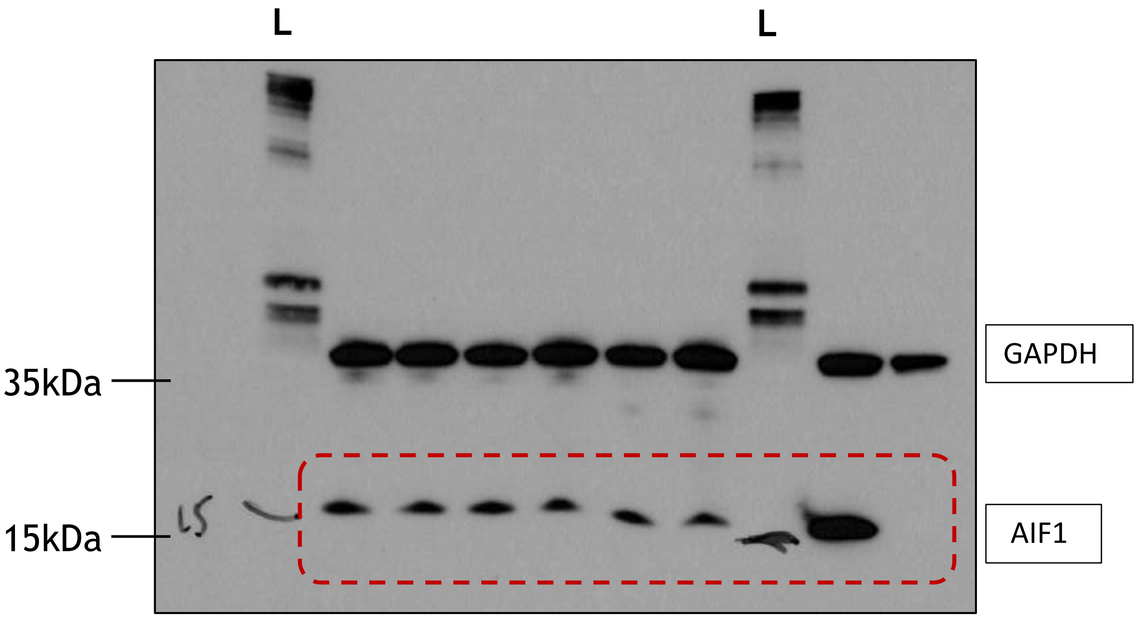

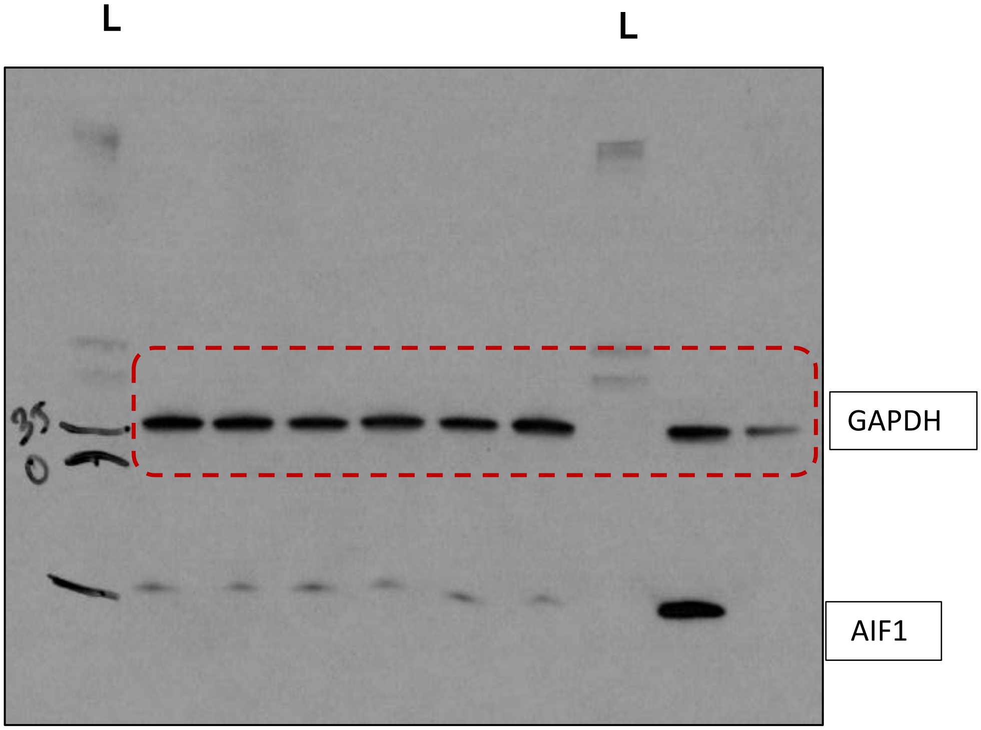

Cropped along the dotted lines

L Ladder

Uncropped blots for Supplementary Figure 4a- Liver

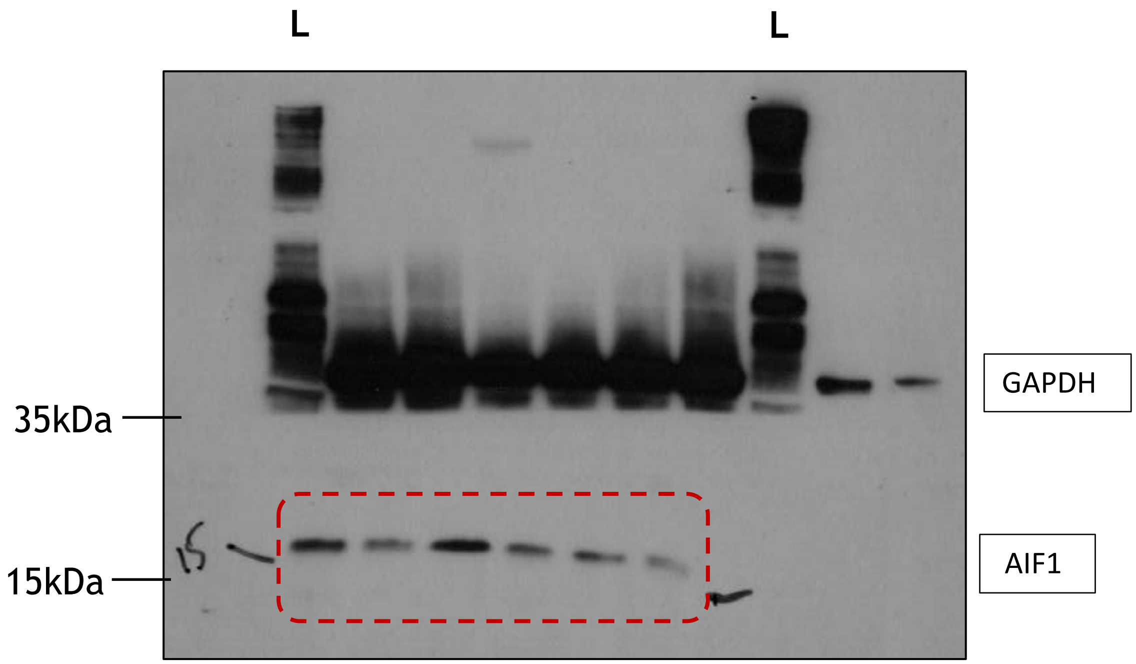

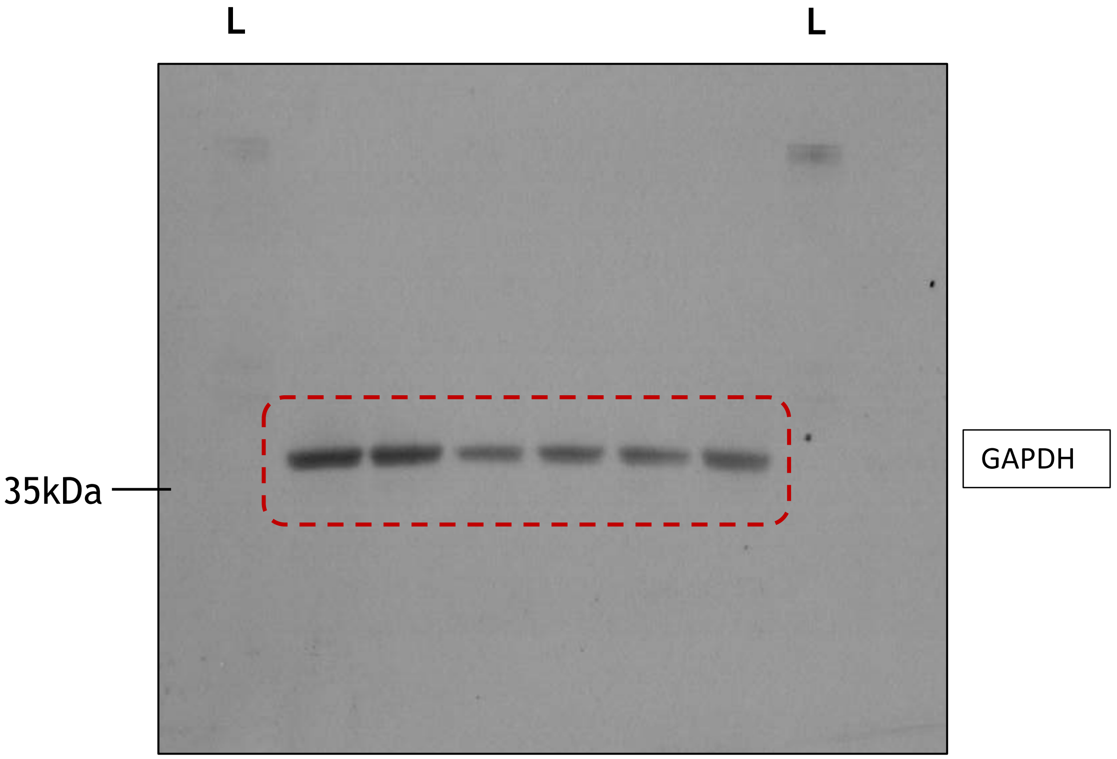

Cropped along the dotted lines

L Ladder

Uncropped blots for Supplementary Figure 4a- Spleen

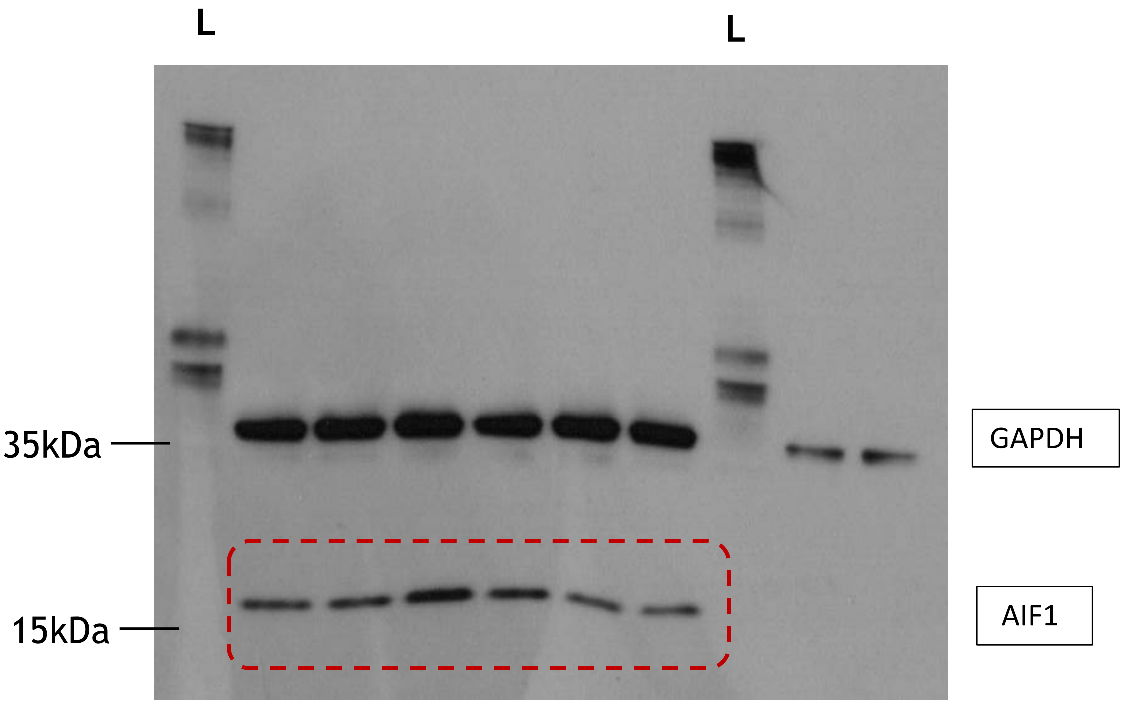

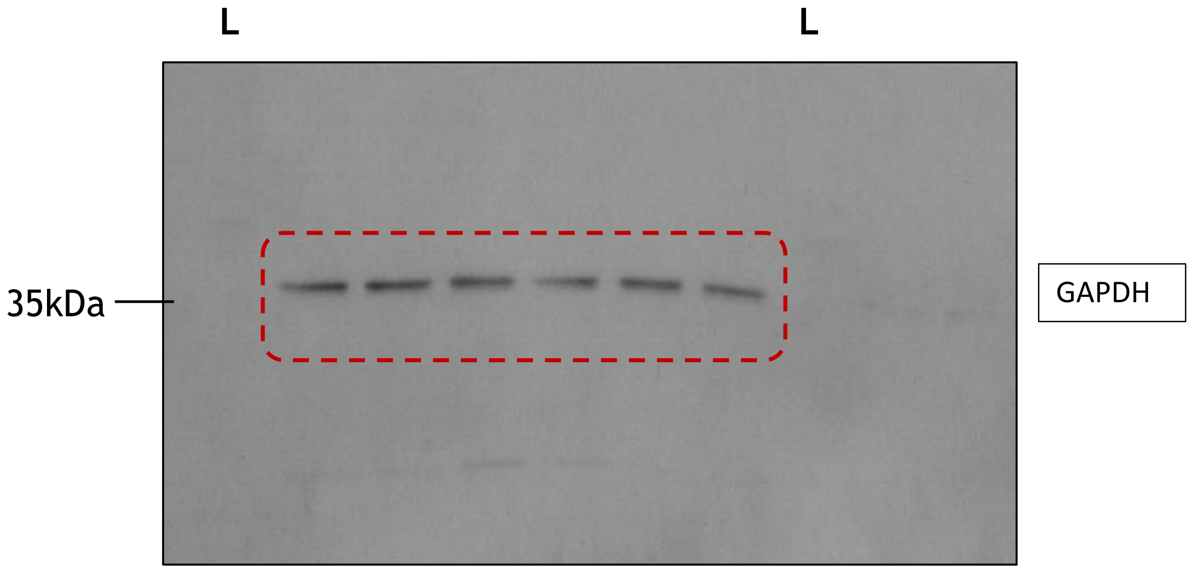

Cropped along the dotted lines

L Ladder
